# The Global Prevalence of Tuberculosis Infection in Buffaloes: A Systematic Review and Meta-Analysis

**DOI:** 10.3390/ani16172774

**Published:** 2026-09-03

**Authors:** Tian Tian, Wei Zheng, Zi-Tong Jing, Qi Wang, Xiao-Nan Wang, Yu-Hao Song, Qing-Long Gong, Dan Yu, Kun Shi

**Affiliations:** 1 College of Veterinary Medicine, Jilin Agricultural University, Changchun 130118, China; 2 Jilin Provincial Engineering Research Center for Sika Deer Efficient Breeding and Product Development Technology, Jilin Agricultural University, Changchun 130118, China; 3 Modern Agricultural College, Changchun Polytechnic University, Changchun 130033, China; 4 Comprehensive Agricultural Law Enforcement Brigade of Dongfeng County, Liaoyuan 136300, China; 5 College of Chinese Medicinal Materials, Jilin Agricultural University, Changchun 130118, China

**Keywords:** buffalo tuberculosis, *Mycobacterium bovis*, prevalence, systematic review, meta-analysis, One Health

## Abstract

**Simple Summary:**

Bovine tuberculosis is a serious disease that can spread between animals and humans, often through unpasteurized milk. Although buffaloes are increasingly recognized as important carriers, no global synthesis of buffalo TB epidemiology has been conducted. This systematic review and meta-analysis of 104 studies, encompassing 385,616 buffaloes across 17 countries over the past 60 years, estimates a global pooled prevalence of 7.4% for buffaloes. However, prevalence varied widely across studies, so this figure should be viewed as a general benchmark rather than a single universal rate. Africa bore the highest burden at approximately 10%. Higher infection rates were associated with factors such as older age, free-ranging management, large herd size, and low national income, with tropical savanna climates also contributing to elevated risk. These findings provide the first global baseline for buffalo tuberculosis and highlight the urgent need for governments and health organizations to include buffaloes in routine surveillance and control programs, especially in regions where raw buffalo milk is consumed and people live in close contact with livestock.

**Abstract:**

Bovine tuberculosis (bTB), caused by *Mycobacterium bovis* (*M. bovis*), is a chronic zoonotic disease with buffaloes as maintenance hosts, yet global pooled prevalence of bTB in buffaloes has not been systematically quantified. This systematic review and meta-analysis estimated the global prevalence, distribution, and risk factors (spatio-temporal, geographical and socioeconomic, host characteristics, and research design and methodology) of bTB in buffaloes. Six electronic databases (PubMed, ScienceDirect, Web of Science, CNKI, VIP, and WanFang) were systematically searched for studies published between 1963 and 2025. Of 1731 records, 104 studies (385,616 buffaloes, 17 countries) were included. Estimates a global pooled prevalence of 7.4% for buffaloes, which is higher than recent global estimates for cattle (reported at 3.98% and, in a 2024 meta-analysis, 3.27% at the animal level); this cross-species comparison should be interpreted cautiously given differences in diagnostics, regions and time periods. Key risk factors identified were advanced age (15–20 years, 34.2%), draught use (28.0%), free-ranging management (12.0%), large herd size (9.9%), low-income economies (13.7%), and tropical savanna climate (10.1%). Publication bias was detected but did not substantially alter the overall pooled prevalence estimate. The persistently high burden in Africa and developing countries highlights the urgent need for integrated One Health surveillance and targeted control strategies.

## 1. Introduction

bTB is a chronic, debilitating, and highly contagious zoonotic disease primarily caused by *M. bovis*, a member of the *Mycobacterium tuberculosis* (*M. tuberculosis*) complex (MTBC) [1,2]. The disease affects a wide range of domestic and wild animal species, as well as humans, and is recognized as a major global public health and economic concern [3,4]. In addition to domestic cattle, numerous other livestock species and wildlife, including buffaloes, deer, badgers, and wild boar [5], are susceptible to *M. bovis* infection, sustaining complex multi-host transmission dynamics that complicate control efforts worldwide [6,7].

Buffaloes represent one of the world’s most economically important livestock species, with a global population exceeding 200 million animals concentrated primarily in Asia, Africa, and South America [8,9]. Although buffalo data have been included incidentally within cattle-centred and multi-species bTB syntheses [10,11,12], to our knowledge, no prior systematic review or meta-analysis has been dedicated specifically to quantifying the global prevalence of TB in buffaloes as the primary target species or to systematically identifying the associated multidimensional risk factors. Their products, including milk, meat, and draught power, are central to the livelihoods of hundreds of millions of smallholder farmers in low- and middle-income countries [13,14]. Beyond their economic significance, buffaloes are increasingly recognized as natural maintenance hosts for *M. bovis*, capable of sustaining and amplifying infection within and across species boundaries [15]. Both domestic water buffaloes (*Bubalus bubalis*) and African buffaloes (*Syncerus caffer*) have been documented as important reservoirs of bTB, with the latter playing a particularly critical role in the wildlife-livestock interface dynamics of sub-Saharan Africa [15,16,17,18]. This interface also has conservation relevance. In Kruger National Park, *M. bovis* infection in lions followed a geo-graphic pattern similar to infection in buffaloes, but ingestion of infected prey has not been proven as the sole route and population-level effects on lions remain uncertain [19,20].

Despite significant progress in bTB control in high-income countries, where systematic test-and-slaughter programs have achieved Officially Tuberculosis-Free (OTF) status in many jurisdictions, the disease remains endemic across Asia, Africa, and South America, where the majority of the world’s buffalo population is concentrated [3,21]. In these regions, inadequate veterinary infrastructure, limited diagnostic capacity, and the absence of national control programs perpetuate high infection rates in both domestic and wild buffalo populations [3,22]. The economic consequences are substantial: bTB causes an estimated 10% reduction in milk yield and 5% reduction in meat production in affected animals, alongside additional losses from trade restrictions, carcass condemnation, and the costs of surveillance and control [23,24]. Beyond direct productivity losses, the zoonotic dimension of buffalo TB, particularly the risk of *M. bovis* transmission to humans through unpasteurized milk consumption [25,26] and occupational exposure [27,28], represents a critical but often overlooked public health burden in endemic regions.

Despite the recognized epidemiological importance of buffaloes as *M. bovis* maintenance hosts, no prior systematic review or meta-analysis has comprehensively quantified the global prevalence of TB in buffaloes or systematically identified the multidimensional risk factors associated with its occurrence. Existing epidemiological data are fragmented across individual country-level studies, conducted with heterogeneous diagnostic methods and reporting standards, making cross-study comparisons unreliable. This evidence gap impedes the development of evidence-based surveillance protocols, risk-based targeting of high-risk populations, and the effective integration of buffalo TB control into One Health frameworks. To address this gap, we conducted the first global systematic review and meta-analysis of buffalo TB prevalence, synthesizing data from 104 studies across 17 countries and five continents, and analyzing risk factors across four dimensions: spatio-temporal, geographic/socioeconomic, host-related, and methodological. The findings of this meta-analysis will establish a comprehensive epidemiological baseline to guide surveillance design, diagnostic selection, and targeted control strategies for buffalo TB worldwide.

## 2. Methods

### 2.1. Search Strategy

This systematic review conforms to Preferred Reporting Items for Systematic Reviews and Meta-analyses (PRISMA) guidelines (Supplementary Table S1) [29]. A comprehensive literature search was conducted across six electronic databases: PubMed, ScienceDirect, Web of Science, CNKI, VIP, and WanFang, covering publications from 1963 to 2025. In PubMed, MeSH terms for “Tuberculosis” and “Buffaloes” were combined using Boolean operators. A representative PubMed search combined MeSH and free-text terms for “Buffaloes”/“Bubalus”/“Syncerus”/“Water Buffalo” with terms for “Tuberculosis”/“Mycobacterium tuberculosis/bovis”; the complete, reproducible search strings for all six databases are provided in the Supplementary Materials. Scopus was not searched owing to institutional access constraints at the time of the search, and the review was not prospectively registered; both points are acknowledged as limitations. In ScienceDirect and Web of Science, the keywords “tuberculosis,” “buffalo,” and “prevalence” were used in advanced search mode. Three Chinese databases (CNKI, VIP and WanFang) were searched using equivalent Chinese-language terms. Eligibility was restricted to studies published in English or Chinese. Scopus was not searched owing to institutional access constraints at the time of the search. A dedicated grey-literature search was not conducted; however, theses and conference proceedings retrieved through the database searches or through reference-list screening were eligible for inclusion if they satisfied all eligibility criteria. Full search strings for all six databases are provided in Supplementary Table S2. Reference lists of all eligible studies were manually screened to identify additional relevant publications not captured by database searches.

### 2.2. Inclusion and Exclusion Criteria

Duplicate/overlapping datasets were identified by comparing author names, institutions, locations, sampling periods and sample sizes; where reports described the same animals, the more complete or more recent report was retained. For repeated sampling of the same population, non-overlapping sampling events were treated as separate study effects, while overlapping events were consolidated to prevent double-counting. Diagnostic cut-offs were those reported in the primary studies and were not re-standardised across studies.

Studies were included if they: (1) investigated buffaloes as the primary study population; (2) used a cross-sectional study design; (3) reported the total number of animals tested and the number of positive cases, enabling prevalence calculation; and (4) were published in English or Chinese with full-text availability. Studies were excluded if they: (1) reported non-tuberculous mycobacterial infections as the primary pathogen; (2) were review articles; (3) employed non-cross-sectional designs; (4) had inaccessible full texts; (5) contained duplicate data; (6) did not meet the species criteria for buffaloes; or (7) had insufficient data for meta-analysis.

### 2.3. Data Extraction

Data were independently extracted by two reviewers using a standardized extraction form in Microsoft Excel 2021. Extracted variables included: first author, publication year, sampling year, country, geographic coordinates, climate type, detection method, sample type, number of animals tested, number of positive cases, host characteristics (age, gender, body condition score (BCS), weight, milk yield, parity, herd size, purpose, source), and study quality score. Discrepancies between reviewers were resolved by consensus. “National income level was assigned to each study’s country using the World Bank Country and Lending Groups income classification (low, lower-middle, upper-middle and high income) (https://datahelpdesk.worldbank.org/knowledgebase/articles/906519, accessed on 20 May 2026), rather than being taken from the primary studies. Geographic coordinates (longitude and latitude) were extracted from the primary study when reported; where coordinates were not reported, approximate coordinates were assigned from the named study location using a mapping service. Because coordinates were unavailable for some studies, the spatial and climate analyses are treated as exploratory. Body condition score was not recorded on a single standardised numerical scale in the primary studies; body condition was therefore extracted as reported and harmonised into two categories (“good” and “poor”), and this variable is treated as exploratory.

### 2.4. Quality Assessment

Study reporting quality was assessed using an author-developed five-point reporting-quality score evaluating: (1) random sampling, (2) explicit description of the diagnostic method, (3) detailed diagnostic methodology, (4) clear specification of the sampling period, and (5) reporting of four or more of these criteria. This score reflects the completeness and transparency of reporting rather than the certainty of the overall body of evidence; the same scheme has been applied in previous animal-disease prevalence meta-analyses [30,31,32,33,34,35]. Scoring was performed independently by two reviewers, with disagreements resolved by discussion. This scoring system evaluated individual study reporting quality and methodological rigor rather than the strength of the overall body of evidence. Studies scoring 0–1 were classified as low quality, 2–3 as moderate quality, and 4–5 as high quality.

### 2.5. Statistical Analysis

All analyses were performed in R (version 3.5.2) using the meta package [36]. The specific R coding process is provided in Supplementary Table S2. Normality of raw prevalence data was assessed using the Shapiro–Wilk test across five transformation methods (raw, logarithmic, logit, arcsine, and double arcsine/Freeman-Tukey) [37]. The Freeman-Tukey double-arcsine transformation (PFT) gave the best-fitting distribution (W = 0.958) and stabilises the variance of proportions near zero; we note that normality is not a formal requirement and that this transformation has known limitations for very small studies and back-transformation, so the pooled value is interpreted as a variance-stabilised summary (Table 1). Heterogeneity was quantified using Cochran’s Q, the I2 statistic, and tau2 (between-study variance) with the chi-square test, and a 95% prediction interval was calculated for the pooled estimate. Univariate subgroup and meta-regression analyses were performed as exploratory analyses; multivariable meta-regression was not undertaken because covariates were incompletely reported and collinear. Publication bias was assessed using funnel plots, Egger’s linear regression test, and the trim-and-fill method. Sensitivity analysis was conducted by sequentially excluding individual studies. Subgroup analyses and univariate meta-regression were performed for all identified risk factors across four analytical dimensions (spatio-temporal, geographical and socioeconomic, host characteristics, and research design and methodology).

## 3. Results

### 3.1. Characteristics of Included Studies

**Figure 1:**
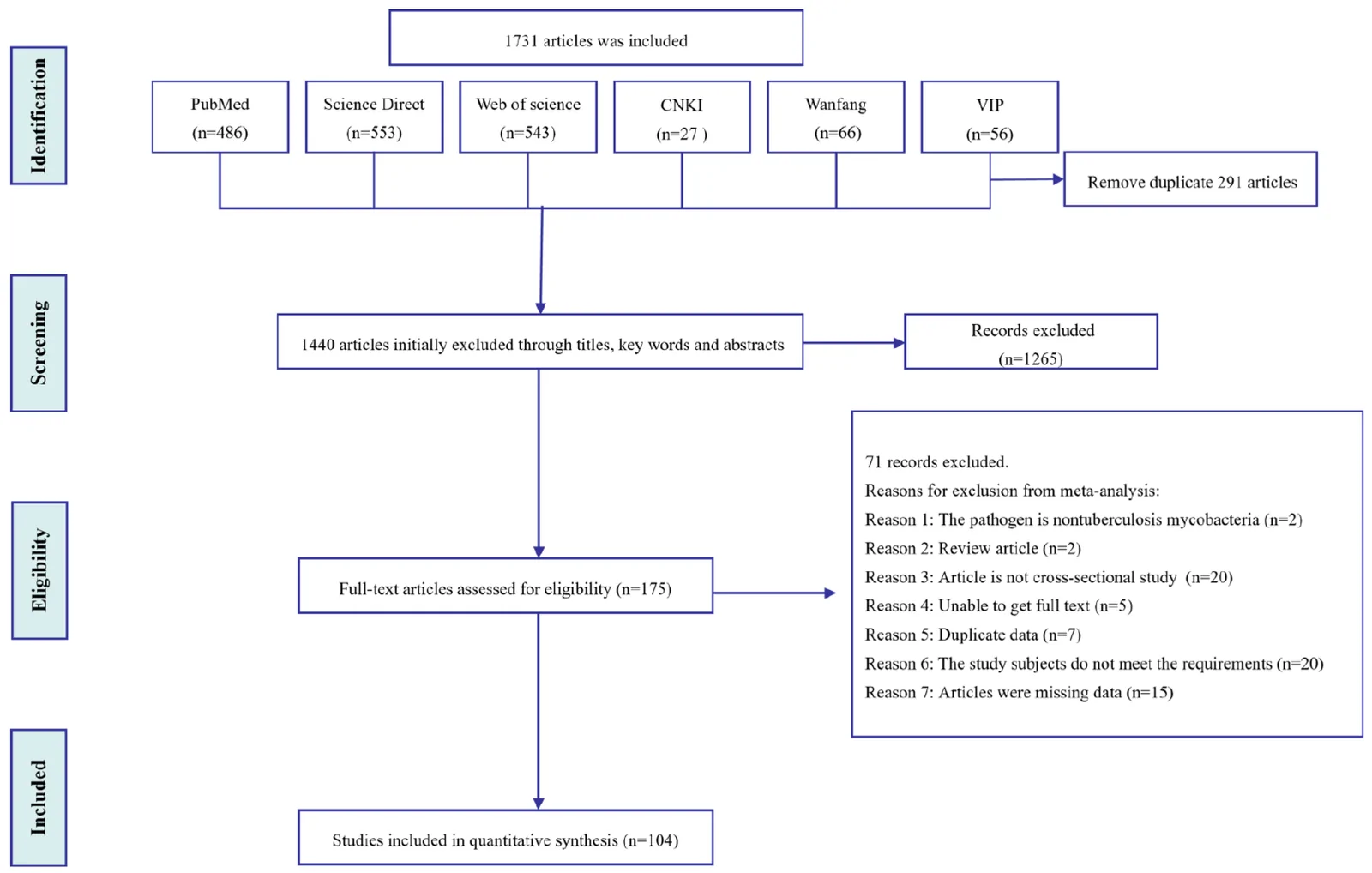
Flow diagram with the four standard stages. In total, 1731 records identified from the six databases, 291 duplicates removed, 1440 records screened, 1265 records excluded at title/abstract screening, 175 reports assessed for eligibility, 71 reports excluded with reasons, and 104 studies included. The per-database record counts and the itemised reasons for full-text exclusion are shown in the diagram.

The systematic database search identified 1731 initial records. After removing 291 duplicates and excluding 1440 records based on title, keyword, and abstract screening, 175 full-text articles were assessed for eligibility. Following full-text review against inclusion and exclusion criteria, 104 studies were included in the final meta-analysis (Figure 1). 

Included studies originated from 17 countries across five continents: Africa (Botswana, Egypt, Mozambique, South Africa, Tanzania, Uganda, Zambia, Zimbabwe), Asia (China, India, Lao PDR, Nepal, Pakistan, Thailand), South America (Brazil), Europe (Italy), and Oceania (Australia). Based on the quality assessment scoring system, 44 studies were classified as moderate quality (2–3 points) and 60 as high quality (4–5 points) (Table 2, Supplementary Table S3).

### 3.2. Heterogeneity Assessment

**Figure 2:**
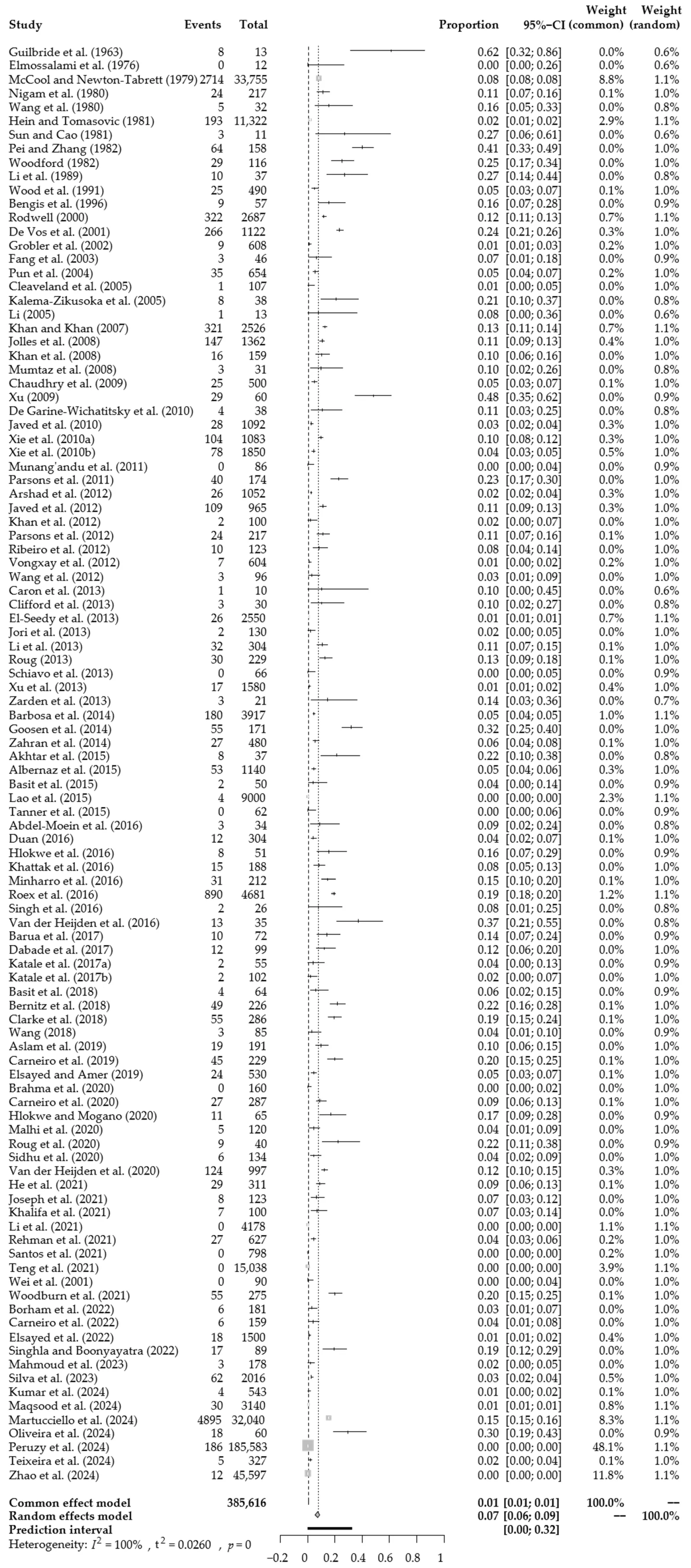
Random-effects model forest plot of global pooled prevalence of buffalo TB (%, 95% CI), with between-study heterogeneity (I^2^ and Q-test *p*-value). Each horizontal line represents the 95% confidence interval of an individual study. The central square indicates the point estimate, with its size reflecting the study’s weight in the pooled analysis. The vertical dashed line at 0 represents the null effect line for proportions. The diamond at the bottom represents the pooled overall estimate, with its width indicating the 95% CI of the combined result. Individual studies are arranged alphabetically by first-author surname [2,38,39,40,41,42,43,44,45,46,47,48,49,50,51,52,53,54,55,56,57,58,59,60,61,62,63,64,65,66,67,68,69,70,71,72,73,74,75,76,77,78,79,80,81,82,83,84,85,86,87,88,89,90,91,92,93,94,95,96,97,98,99,100,101,102,103,104,105,106,107,108,109,110,111,112,113,114,115,116,117,118,119,120,121,122,123,124,125,126,127,128,129,130,131,132,133,134,135,136,137,138,139,140].

Forest plot analysis revealed high heterogeneity across included studies (I^2^ = 99.7%, *p* < 0.001) (Figure 2). The wide dispersion of individual study effect sizes and minimal overlap of confidence intervals confirmed substantial between-study variation. Given this high heterogeneity, a random-effects model was applied for all pooled estimates, and subsequent subgroup analyses and meta-regression were conducted to explore sources of heterogeneity.

### 3.3. Evaluation of Publication Bias

Funnel plot analysis revealed marked asymmetry (Figure 3), with sparse and uneven distribution of study points in the region of small effect sizes (prevalence approaching zero), suggesting the presence of publication bias, likely attributable to underrepresentation of small-sample studies with low or zero prevalence. Egger’s linear regression test confirmed significant publication bias (*p =* 0.0003) (Figure 4, Supplementary Table S4). While the trim-and-fill analysis imputed additional studies to correct for asymmetry (Figure 5); the adjusted pooled prevalence remained consistent with the unadjusted estimate, confirming the robustness of our overall findings. Funnel plots for each subgroup are presented in Supplementary Figures S1–S24.

### 3.4. Sensitivity Analysis

**Figure 6:**
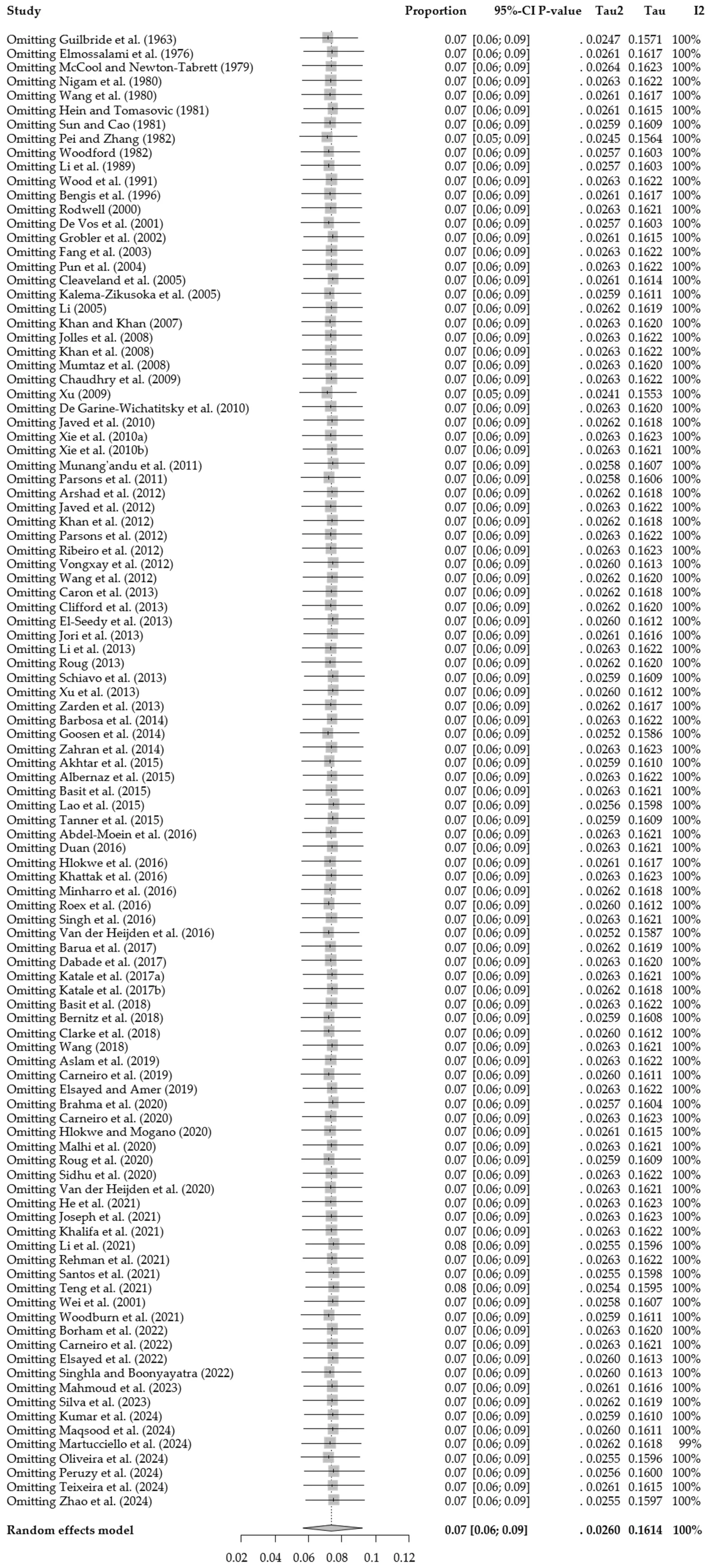
Leave-one-out sensitivity analysis. Each black dot represents the pooled prevalence estimate recalculated after sequentially removing one study. The horizontal line extending from each dot represents the 95% confidence interval of that estimate. The vertical dashed line indicates the overall pooled prevalence estimate from all studies combined. The diamond at the bottom represents the overall pooled estimate with its 95% confidence interval [2,38,39,40,41,42,43,44,45,46,47,48,49,50,51,52,53,54,55,56,57,58,59,60,61,62,63,64,65,66,67,68,69,70,71,72,73,74,75,76,77,78,79,80,81,82,83,84,85,86,87,88,89,90,91,92,93,94,95,96,97,98,99,100,101,102,103,104,105,106,107,108,109,110,111,112,113,114,115,116,117,118,119,120,121,122,123,124,125,126,127,128,129,130,131,132,133,134,135,136,137,138,139,140].

Sensitivity analysis, performed by sequentially excluding individual studies, demonstrated that the pooled prevalence estimate and its 95% confidence interval remained stable, with no single study exerting undue influence on the overall result. The I^2^ and τ^2^ statistics also remained consistently high across all iterations, indicating that the observed heterogeneity was not driven by any single influential study (Figure 6).

### 3.5. Meta-Analysis of Buffalo Tuberculosis

The global pooled prevalence of buffalo TB across all 104 included studies was 7.4% (95% CI: 5.7–9.2), derived from 385,616 animals tested and 11,917 confirmed positive cases (Table 3 and Supplementary Table S5). Country-level prevalence estimates varied widely, ranging from 1.5% (Botswana) to 32.0% (Uganda) (Table 4).

### 3.6. Risk Factors Associated with Buffalo Tuberculosis

Given the high heterogeneity, subgroup analyses and univariate meta-regression were conducted to explore sources of variation and identify risk factors. Comprehensive results are detailed in Table 3.

#### 3.6.1. Host Characteristics

BCS, gender, age, body weight, milk yield, parity, herd size, purpose, and source were classified as host characteristic variables. Poor BCS showed a numerically higher prevalence (33.3%, 95% CI: 0.1–99.8) compared to good BCS (4.5%, 95% CI: 0.4–11.3), though the difference was not statistically significant (*p* = 0.166). No statistically significant difference in prevalence was observed between female and male buffaloes (5.7%, 95% CI: 3.3–8.5 vs. 3.5%, 95% CI: 0.0–12.6; *p* = 0.415). Age showed a significant positive association with prevalence (*p =* 0.018), with the 15–20 year group showing the highest prevalence (34.2%, 95% CI: 2.6–75.4; *n* = 30 animals, 3 studies) compared to <1 year (0.0%), 1–5 years (0.5%), and 5–15 years (11.7%). Body weight > 500 kg was associated with higher prevalence (9.9%) than ≤500 kg (3.4%; *p* = 0.047). Prevalence appeared to increase with milk yield (from 1.5% in <5 L to 9.3% in >15 L), but this trend was not statistically significant (*p* = 0.450). Buffaloes with 5–10 parities showed higher prevalence (11.0%) than those with <5 parities (4.6%), but the difference was not statistically significant (*p* = 0.152). Herd size of 100–1000 animals was associated with significantly higher prevalence (10.3%) than herds < 100 animals (0.0%; *p* < 0.001). Draught buffaloes showed the highest prevalence by purpose (28.0%, 95% CI: 15.3–42.6; *n* = 219 animals, 4 studies), followed by wild buffaloes (12.7%) and dairy buffaloes (4.1%; *p* < 0.001). Free–ranging animals showed higher prevalence (12.0%) than farm-housed (5.9%) and abattoir-sourced animals (3.5%; *p* = 0.001).

#### 3.6.2. Research Design and Methodology

Detection method, tuberculin test classification, sample type, mycobacterial species, and study quality were classified as research design and methodology variables. Among detection methods, ZN staining showed the highest prevalence (10.1%), followed by IFN-γ ELISA (9.1%), post-mortem examination (8.3%), TST (7.8%), PCR (6.8%), TB ELISA (6.2%), and culture (5.7%); differences were not statistically significant (*p* = 0.577). The pooled prevalence was numerically higher with CITT (9.9%) than with SITT (6.1%), but this difference was not statistically significant (*p* = 0.133). Blood samples showed the highest prevalence (9.8%), while TB-like lesions showed the lowest (1.3%; *p* = 0.011). *M. bovis* was the most commonly identified species (7.4%), followed by *M. tuberculosis* (3.5%) and *M. orygis* (3.3%); differences were not statistically significant (*p* = 0.545). Study quality did not significantly influence prevalence estimates (*p* = 0.719).

#### 3.6.3. Geographical and Socioeconomic Factors

Region, sub-region, development status, and income level were classified as geographical and socioeconomic variables. Africa showed the highest regional prevalence (10.1%, 95% CI: 6.9–13.7; *p* = 0.022), with Eastern Africa (13.5%) and Southern Africa (13.0%) showing the highest sub-regional estimates. Europe showed the lowest prevalence (2.6%). Income level was a significant predictor (*p* = 0.032): low-income economies showed the highest prevalence (13.7%), followed by upper-middle-income (9.7%), lower-middle-income (4.8%), and high-income economies (3.6%).

#### 3.6.4. Spatio-Temporal Factors

Publication year, sampling year, season, longitude, latitude, and climate type were classified as spatio-temporal variables. Both publication year (*p* = 0.005) and sampling year (*p* = 0.006) showed significant negative associations with prevalence, with the highest prevalence before 2000 (15.8% and 12.8%, respectively) declining to the lowest in 2020–2025 (4.5% and 1.5%, respectively). Dry season prevalence (11.8%) exceeded rainy season prevalence (4.0%), though the difference was not statistically significant (*p* = 0.438). Longitude (*p* = 0.001) and latitude (*p* < 0.001) were significant predictors, with the 30–60° E band and 20–30° S latitude zone showing the highest prevalence. Tropical savanna climate was associated with the highest prevalence (10.1%; *p* < 0.001) compared to Mediterranean (3.2%) and other climate types.

## 4. Discussion

This study represents the first comprehensive systematic review and meta-analysis to quantify the global prevalence of tuberculosis in buffaloes. The pooled global prevalence of buffalo TB was estimated at 7.4%, establishing a critical epidemiological baseline for this underappreciated host species. This population-level baseline fills a major global prevention gap, as prior bTB control frameworks have long centred exclusively on cattle and ignored buffalo populations across multi-continental production systems. This estimate is notably higher than the pooled global prevalence of bTB in cattle, which has been reported at 4.0% in a recent global meta-analysis [10], and substantially higher than bTB prevalence in dairy cattle in China at 2.4% [141] and in India at 7.3% [142]. In contrast, TB prevalence in wild boar has been estimated at 21.98% and in red deer at 13.7% globally [6], while swine TB stands at 12.1% [143], suggesting that buffaloes occupy an intermediate epidemiological position among susceptible livestock and wildlife species. This intermediate risk profile renders buffaloes a critical bridging reservoir linking high-prevalence wild ungulates and low-risk domestic cattle, creating persistent spillover risks that cannot be overlooked in cross-species disease prevention schemes. The comparatively elevated prevalence in buffaloes relative to cattle likely reflects the dual role of buffaloes as both maintenance hosts for *M. bovis* [144,145] and animals frequently managed under conditions that amplify transmission risk, including extensive grazing systems [43], mixed-species herding [144], and limited veterinary oversight in endemic regions [146]. From a disease control perspective, this elevated prevalence signals that buffaloes should no longer be overlooked in national bTB control strategies; instead, they must be explicitly incorporated as a priority target population in surveillance and intervention programs, particularly in regions where buffalo production is economically significant. The observed extreme heterogeneity across included studies is not unexpected in a global meta-analysis of this scope and reflects genuine biological and methodological variation rather than statistical artifact [147]. This level of heterogeneity is consistent with other large-scale bTB meta-analyses [10] and underscores the profound influence of geographic, ecological, socioeconomic, and methodological factors on observed prevalence estimates. Therefore, the use of a random-effects model and the PFT is appropriate and methodologically justified [37]. This high heterogeneity indicates that a single global prevalence estimate, while useful as a summary statistic, should be interpreted alongside the subgroup analyses that reveal the true heterogeneity of the epidemic across different contexts [148,149]. Because the heterogeneity was extreme (I^2^ approaching 100%), the pooled estimate should be regarded as a descriptive central tendency rather than a value that applies to any single setting; the subgroup and meta-regression results, not the global figure, carry the epidemiologically meaningful signal. The individual covariates examined here each account for only part of the between-study variance, and their combined contribution was not jointly modelled; a large fraction of the heterogeneity therefore remains unexplained and is likely driven by unmeasured differences in diagnostic protocols, test cut-offs, sampling frames and host populations.

Publication bias was detected in this study, suggesting that small-sample studies with low or zero prevalence may be underrepresented in the published literature [150,151]. This is a common limitation in prevalence meta-analyses and is likely associated with a modest overestimation of the true pooled prevalence [151]. However, the trim-and-fill analysis confirmed that the overall direction and magnitude of the pooled estimate remained robust after correction, thereby supporting the validity of our findings [152,153].

The geographic distribution of buffalo TB prevalence revealed a clear gradient, with higher prevalence in Africa and South America and the lowest in Europe. This pattern closely mirrors the global distribution of bTB in cattle, where Africa and Asia bear the greatest burden [10,154], and reflects the convergence of ecological, socioeconomic, and policy-related determinants [147]. When explaining these regional differences in prevalence, it is essential to consider the imbalance across continents in terms of the number of studies and the size of animal samples. The current evidence base is primarily concentrated in Africa and Asia, while Europe and Oceania have contributed only a handful of studies, and some major buffalo-producing regions are entirely absent; therefore, prevalence estimates for regions with limited research are subject to considerable uncertainty. Furthermore, control methods vary significantly across regions: ranging from systematic “test-and-slaughter” programs implemented in high-income countries, to targeted control activities for native water buffalo in Italy, to the complex environment in Southern Africa where wild animals serve as multi-host reservoirs (in these regions, relying solely on livestock control measures often yields limited results). The extent to which control measures are implemented ultimately depends on each country’s legislative framework and the capacity of its veterinary services. Critically, this stark disparity underscores the decisive role of institutional capacity, economic investment, and sustained political will in disease control outcomes. The high prevalence in Africa, particularly in regions with extensive wildlife-livestock interfaces, highlights an urgent need for targeted surveillance and control programs that are adapted to local ecological and socioeconomic contexts. Within Africa, the highest sub-regional prevalence was recorded in Eastern and Southern Africa. These regions are characterized by extensive wildlife-livestock interfaces, particularly in and around protected areas such as Kruger National Park (South Africa) [42,43] and the Serengeti ecosystem (Tanzania) [45,64], where African buffaloes (*Syncerus caffer*) serve as established maintenance hosts for *M. bovis* and act as a persistent reservoir for spillover into domestic livestock [64]. The epidemiological dynamics at these interfaces are complex: indirect interactions between wildlife and cattle, mediated by shared water sources [155,156], grazing areas [157], and environmental contamination [156,158], have been shown to occur at rates 154-fold higher than direct contact events, highlighting a plausible role for environmental transmission that warrants further study [7]. For field prevention, this finding highlights shared waterpoints as high-priority intervention sites, where regular environmental disinfection and rotational grazing separation between wildlife and domestic buffaloes could substantially cut indirect transmission chains. The high prevalence in Southern Africa is further compounded by the presence of large free-ranging buffalo populations in game reserves, where culling-based control is ethically and practically constrained [3,70,159]. In such contexts, alternative interventions—including wildlife vaccination, improved biosecurity at interface areas, and community-based surveillance—should be explored and evaluated as complementary strategies to conventional test-and-slaughter approaches. In contrast, the remarkably low prevalence in Europe, derived entirely from studies conducted in Italy, reflects the impact of sustained, well-resourced eradication programs [125]. Italy has implemented mandatory tuberculin skin testing, movement restrictions, and slaughter of reactor animals in Mediterranean water buffalo (*Bubalus bubalis*) herds for over a decade, with documented reductions in herd-level prevalence [125]. This Italian experience provides a compelling proof-of-concept that buffalo TB can be substantially reduced through systematic surveillance and test-and-slaughter policies, even in high-density dairy buffalo systems. As such, the Italian model offers a valuable template for other endemic regions, demonstrating that sustained political commitment and adequate resourcing can yield measurable progress even in complex production systems. The stark contrast between European and African prevalence underscores the decisive role of institutional capacity and political will in disease control outcomes [3]. Such geographic disparities are further mirrored at the national economic level. The strong inverse association between national income level and buffalo TB prevalence reflects the structural inequities in global animal health systems. This pattern mirrors the global distribution of human TB, where 95% of TB deaths occur in low- and middle-income countries [160], and reflects the convergence of multiple socioeconomic disadvantages: limited veterinary infrastructure, inadequate diagnostic capacity, absence of national control programs, poor biosecurity practices, and low awareness of zoonotic risks among livestock producers and consumers [161]. The finding that landlocked developing countries had a higher prevalence suggests a potential role of geographic isolation in shaping disease burden. This may be because landlocked status limits access to international veterinary expertise, diagnostic reagents, and pharmaceutical inputs, while also constraining the ability to implement movement controls and trade-based incentives for disease control. Countries such as Uganda, Zambia, and Zimbabwe, which are substantially associated with the African dataset, exemplify these challenges. Addressing these structural inequities will require concerted international support, including technology transfer, capacity building, and targeted funding for veterinary infrastructure in low-income countries. The knowledge, attitudes, and practices (KAP) dimension of bTB control in low-and middle-income countries deserves particular attention. A systematic review found that awareness of bTB and its zoonotic potential is surprisingly low among African and Asian livestock producers and consumers, even in high-exposure settings [22]. This knowledge gap perpetuates risky practices such as consumption of raw milk, inadequate hand hygiene after animal contact, and failure to report sick animals to veterinary authorities. This finding has direct implications for intervention design: technical surveillance measures alone will be insufficient without parallel investments in community education and behavior change communication. Addressing these behavioral determinants through targeted One Health education campaigns is an essential complement to technical surveillance and control measures. For national prevention authorities, KAP interventions should be embedded into routine livestock extension services to achieve long-term reduction in zoonotic transmission risks without excessive additional fiscal costs.

From a climatic and ecological perspective, the climate analysis showed that tropical savanna climates were associated with the highest prevalence, significantly higher than Mediterranean climates. This association is ecologically plausible. *M. bovis* can survive in the environment for weeks to months under cool [162,163], moist conditions, but the combination of seasonal water scarcity in savanna ecosystems forces wildlife and livestock to congregate at shared water points [155,156,157], dramatically increasing transmission opportunities. Our analysis showed a numerically higher prevalence in the dry season than in the rainy season, although this difference was not statistically significant. This numerical pattern is consistent with the hypothesized mechanism that dry-season congregation behavior amplifies both direct and indirect contact rates among susceptible hosts, but should be interpreted with caution given the lack of statistical support [155,157,164]. This seasonal disparity provides a clear seasonal prevention schedule: intensified targeted screening, waterpoint sanitation, and restricted mixed grazing should be scheduled uniformly across dry seasons in tropical savanna zones to interrupt seasonal transmission peaks. The latitude analysis further supports this interpretation. The 20–30° S latitude band, which encompasses the core of the Southern African savanna and game reserve belt, showed the highest prevalence, while tropical and subtropical zones with more dispersed animal distributions showed lower rates [10,165]. This spatial concentration of high prevalence provides a clear geographic targeting opportunity for resource allocation: surveillance programs can prioritize these high-risk latitudinal bands, particularly in Southern Africa, where the convergence of ecological and management factors creates persistently elevated transmission risk.

Temporal trend analysis revealed a consistent and significant decline in the prevalence of buffalo tuberculosis. Subgroup analyses based on publication year and sampling year both showed that prevalence declined from higher levels before 2000 to lower levels during 2020–2025. Meta-regression confirmed a significant negative association between time and prevalence. Because the included evidence is cross-sectional, this pattern is most consistent with changes in measured prevalence, rather than incidence. This temporal decline likely reflects the cumulative effect of multiple converging factors [3]. A key contributor is the global expansion of national bTB control programs, including mandatory testing, movement controls, and slaughter compensation schemes, which has progressively reduced the reservoir of infected animals in many countries [125,166]. This trend is also evident in Europe; for example, the specific control measures implemented for domestic water buffalo in Italy are a typical case in point. Another important factor is that improvements in diagnostic sensitivity and specificity, particularly the wider adoption of IFN-γ release assays (IGRA) [67,107,125,167] and CITT [2,107,119], have enabled more accurate identification and removal of infected animals. This tiered approach is a context-dependent recommendation rather than a universal prescription: the optimal test sequence and control intensity should be adapted to the surveillance objective, local epidemiology, wildlife-reservoir context, and available veterinary and financial capacity. Furthermore, correlated with awareness among veterinary authorities and livestock producers of the zoonotic risks associated with bTB has driven greater investment in surveillance infrastructure [22]. However, to sustain and accelerate this downward trajectory, particularly in high-burden regions, future efforts must prioritize strengthening veterinary capacity, expanding surveillance coverage to under-sampled populations, and ensuring that control programs are adequately resourced and politically supported over the long term. Furthermore, the steeper decline observed in sampling year data compared to publication year data warrants careful interpretation. The sampling year trend showed a marked decline, which may partly reflect a genuine reduction in true prevalence driven by control interventions, but may also be influenced by a shift in study populations: more recent studies tend to be conducted in intensively managed, commercially oriented herds where surveillance is more systematic and control measures are more consistently applied, potentially underrepresenting the true burden in smallholder and free-ranging systems [22,115,116]. Furthermore, the dramatic decline in the most recent sampling period is based on only a small number of studies, and the wide confidence interval reflects substantial residual uncertainty. Caution is therefore warranted in interpreting this as evidence of near-elimination; it may instead reflect sampling bias toward well-managed, low-prevalence settings in recent years [10]. This sampling bias implies that current global downward trends may overestimate the effectiveness of existing prevention policies in low-resource smallholder and pastoral production systems, which remain the dominant buffalo rearing model across Africa and South Asia. Despite the encouraging downward trend, the persistence of high prevalence in sub-Saharan Africa [10,168] and parts of South America [17,142] indicates that global progress remains uneven. This uneven progress underscores the need for geographically differentiated control strategies: in regions with established control programs, efforts should focus on sustaining gains and addressing residual hotspots; in high-burden regions with limited resources, the immediate priority must be building foundational surveillance and diagnostic capacity. When interpreting the above conclusions, several important limitations should be noted. First, the pooled estimates are derived predominantly from studies of domestic water buffalo (Bubalus bubalis) and African buffalo (*Syncerus caffer*) in Africa and Asia; although African buffalo are well represented as wildlife maintenance hosts (19 studies), the evidence base for other wild bovid hosts and for several major buffalo-producing regions is thin, so the pooled estimate should be extrapolated to unstudied populations with caution. Second, the differences in diagnostic methods among studies (post-mortem examination, tuberculin testing and PCR) constitute an important source of bias: these methods differ in sensitivity and specificity, which may partly explain the high heterogeneity observed (I^2^ = 99.7%). Readers should therefore take the effect of diagnostic-method differences into account when interpreting the prevalence estimates. Future work should prioritise standardised diagnostic protocols and harmonised test-interpretation criteria, together with more detailed epidemiological investigation at the wildlife–livestock interface, to clarify the true risk of cross-species transmission.

The subgroup analyses of host characteristics revealed several biologically meaningful patterns that illuminate the immunological and physiological determinants of buffalo TB susceptibility. Given that the available evidence is largely cross-sectional, these findings are best viewed as risk indicators supported by plausible mechanisms, rather than as causal determinants of infection. Age-stratified analysis showed higher prevalence with advancing age, peaking in the 15–20 year age group, whereas prevalence was very low in animals under one year and in the 1–5 year group. This age-dependent pattern is consistent with the epidemiology of bTB in cattle, where older animals show higher prevalence due to cumulative lifetime exposure to *M. bovis* [10]. However, the immunological basis extends beyond simple exposure accumulation. In older animals, progressive immunosenescence, the age-related decline in immune function, is associated with reduced T-cell responsiveness, impaired IFN-γ production, and diminished capacity to maintain granulomatous containment of mycobacterial infection [169]. The Th1-mediated cellular immune response, which is central to mycobacterial control, becomes less robust with age, potentially allowing latent infections to reactivate or progress to active disease [170]. The very high prevalence in the 15–20 year group, although based on a small sample, is consistent with this immunosenescence hypothesis and warrants further investigation in larger longitudinal studies. From a preventive perspective, this age gradient supports the design of age-stratified screening protocols, with annual mandatory surveillance targeting buffalo herds older than 10 years and early culling of infected aged individuals to reduce within-herd pathogen reservoirs. Although BCS has been identified as a risk factor in previous cattle studies [171], our analysis did not detect a statistically significant association in buffaloes, although poor BCS animals showed a numerically higher prevalence than those with good BCS. The observed numerical trend reflects the well-established nutritional-immune axis: chronic undernutrition impairs multiple components of innate and adaptive immunity, including macrophage bactericidal activity, lymphocyte proliferation, and cytokine production [172,173]. In resource-limited settings where buffalo are frequently maintained on marginal nutrition, poor BCS may serve as both a marker of immune vulnerability [174,175] and a modifiable risk factor amenable to nutritional intervention [176]. However, given the lack of statistical significance in our data, these potential implications should be interpreted with caution: nutritional supplementation programs, particularly during periods of feed scarcity, may not only improve animal productivity and welfare but also enhance immunological resilience against bTB. Integrating nutritional support into existing veterinary extension services could therefore yield dual benefits for both production and disease control, but this hypothesis requires further investigation in larger, well-designed studies before being recommended as a specific bTB control measure. Numerically, the prevalence in female buffaloes was higher than in males, although this difference was not statistically significant. This numerical pattern is consistent with observations in cattle and may reflect the immunomodulatory effects of pregnancy and lactation. During gestation, progesterone-mediated immune suppression shifts the immune response toward a Th2 profile, potentially reducing the Th1-mediated protection against intracellular pathogens such as *M. bovis* [170,177]. Additionally, the physiological stress of repeated pregnancies and high milk production may chronically elevate cortisol levels, further suppressing cell-mediated immunity [87,178]. Female buffaloes also tend to have longer productive lifespans and greater cumulative exposure to infected herd-mates, contributing to higher lifetime infection risk [174,175]. Given that this was only a numerical observation without statistical significance, it should not be overinterpreted; however, the higher economic value and longer productive lifespan of female buffaloes may still justify continued attention to this demographic in surveillance programs, particularly during periods of elevated physiological stress such as late gestation and early lactation. The dramatically elevated prevalence in draught buffaloes compared to dairy and wild buffaloes is a particularly striking finding. Draught animals are subjected to chronic physical stress, which activates the hypothalamic–pituitary–adrenal (HPA) axis and elevates circulating glucocorticoids [179,180]. Sustained hypercortisolemia suppresses lymphocyte function [181], reduces IFN-γ production [182], and impairs macrophage activation [183,184], precisely the immune mechanisms required for mycobacterial control. Furthermore, draught buffaloes frequently work in close proximity to other animals and humans, increasing exposure opportunities [175,185]. This finding has direct practical implications: draught buffalo populations should be prioritized as a high-risk group in surveillance programs, and efforts to reduce occupational stress through improved working conditions, adequate rest periods, and workload management may have ancillary benefits for TB control. Given that draught buffaloes are also valuable economic assets for smallholder farmers, investing in their health could simultaneously improve animal welfare and safeguard household livelihoods. Beyond human-mediated working stress, higher prevalence in free-ranging/wild buffaloes compared to farm-housed animals reflects the combined effects of unrestricted movement [159,186], contact with wildlife reservoirs [146,186], absence of veterinary intervention [187], and the ecological dynamics of multi-host transmission systems [3,157,186]. This finding highlights a fundamental challenge for bTB control in regions with extensive wildlife-livestock interfaces: conventional farm-based surveillance and control measures are insufficient to address transmission in free-ranging populations. Alternative approaches, such as wildlife vaccination, enhanced biosecurity at interface zones, and participatory surveillance involving local communities, are needed as complementary strategies. In parallel, transmission dynamics at the population level are also shaped by herd size. The large herd size was also associated with higher prevalence compared to small herds, consistent with density-dependent transmission dynamics where the probability of contact with an infectious individual correlates with herd size [174,188]. For control programs, large commercial and communal herds should implement regular batch testing and partitioned housing to lower intra-herd contact rates and block density-dependent transmission, while interventions in smaller herds may focus on improving early detection and reporting mechanisms. 

The choice of diagnostic method profoundly influences observed prevalence estimates and has critical implications for surveillance program design. These differences reflect the distinct sensitivity and specificity profiles of each method, as well as the populations in which they are applied. The TST remains the most widely used screening tool, consistent with its endorsement by the World Organisation for Animal Health (WOAH) as the standard ante-mortem test for bTB [189]. Its relatively lower apparent prevalence compared to IGRA likely reflects its lower sensitivity in detecting early or subclinical infections, particularly in animals with waning cell-mediated immune responses [189]. The IGRA, which measures IFN-γ release from sensitized T-lymphocytes in response to mycobacterial antigens, offers superior sensitivity for detecting early infection and is less affected by the non-specific reactions that can confound TST interpretation in regions with high environmental mycobacterial exposure [190,191]. The higher IGRA prevalence in our analysis is therefore likely to reflect more accurate detection of true infection rather than false positivity. A further refinement in diagnostic specificity is offered by the CITT. The comparison between CITT and SITT is particularly informative. The CITT, which uses both bovine and avian PPD simultaneously, effectively discriminates *M. bovis* infection from cross-reactions associated with non-tuberculous mycobacteria (NTM) and environmental mycobacterial sensitization [192], which are common in tropical and subtropical environments [193]. The SITT cannot distinguish *M. bovis* sensitisation from cross-reactivity to non-tuberculous and environmental mycobacteria, whereas the comparative CITT is designed to make this distinction; comparative and IFN-gamma-based assays have been reported to outperform the single test in buffaloes [167,194]. The CITT yielded a numerically higher pooled prevalence estimate than the SITT, but this difference was not statistically significant, suggesting that the apparent advantage of CITT over SITT in this context warrants further investigation. In regions with high NTM prevalence, such as tropical Africa and South Asia, the CITT offers particular advantages due to its higher specificity [2]. This subgroup comparison was not adjusted for potential confounders such as region, host species (African buffalo versus domestic water buffalo) and sampling year, and is therefore descriptive rather than an unconfounded estimate of relative test performance. In contrast to ante-mortem diagnostic approaches, post-mortem examinations are subject to important ascertainment biases. The relatively high prevalence detected by PM examination reflects the fact that PM studies are typically conducted in abattoir settings where animals with visible TB-like lesions are selectively sampled, introducing ascertainment bias toward more advanced disease [195]. Conversely, the low prevalence of TB-like lesions in our sample-type analysis suggests that gross pathological examination alone substantially underestimates true infection prevalence, as many infected animals do not develop macroscopically visible lesions, particularly in early or subclinical infection stages [196]. Beyond test type, the choice of biological sample also affects prevalence estimates. Blood-based samples and milk samples showed higher prevalence than serum and tissue samples, supporting the utility of non-invasive ante-mortem sampling for surveillance. The detection of *M. bovis* in milk is of particular public health significance, as it represents a direct route of zoonotic transmission to humans through consumption of unpasteurized dairy products. A meta-analysis of *M. bovis* in dairy cattle milk found an overall prevalence of 5% in individual cow milk samples [26], and our finding in buffalo milk samples suggests that buffaloes may represent an even more significant source of zoonotic exposure through dairy products in endemic regions. Based on these diagnostic considerations, for resource-limited settings, a tiered diagnostic algorithm is recommended: TST or CITT as the primary screening tool, with IGRA confirmation of TST-positive animals to maximize both sensitivity and specificity. This approach, analogous to the two-step testing strategy used in human TB diagnosis [197], would optimize the cost-effectiveness of surveillance while minimizing both false positives (which trigger unnecessary culling) and false negatives (which allow infected animals to remain in herds). Implementing such tiered algorithms in resource-limited settings will require careful consideration of local infrastructure, laboratory capacity, and cost constraints. Pilot studies evaluating the operational feasibility and cost-effectiveness of this approach in different epidemiological contexts should be prioritized as a research agenda. 

The zoonotic significance of buffalo TB extends well beyond animal health and productivity. *M. bovis*, the predominant pathogen in buffalo TB, is capable of infecting humans through multiple routes: inhalation of infectious aerosols from infected animals, consumption of unpasteurized milk and dairy products, and occupational exposure during slaughter, veterinary procedures, and carcass handling. A global meta-analysis estimated that *M. bovis* accounts for 12.1% of all human MTBC isolates when older diagnostic techniques are used, and 1.4% when genotyping-based methods are applied, suggesting that the true global burden of zoonotic TB remains uncertain and likely varies substantially by region and diagnostic capacity [198]. Beyond the well-recognized zoonotic risk from *M. bovis*, our analysis also revealed evidence of reverse zoonosis. The detection of *M. tuberculosis* in buffalo samples in our analysis, while less common than *M. bovis*, raises additional concerns about reverse zoonosis, the transmission of human-adapted mycobacteria to animals, which has been documented in settings where humans and buffaloes share close living spaces [77]. The identification of multidrug-resistant *M. tuberculosis* in buffalo samples from Egypt is particularly alarming, as it suggests that buffaloes may serve as amplifying hosts for drug-resistant strains, with potential implications for both animal and human TB control programs [77]. The public health risk is most acute in settings where buffaloes are managed in close proximity to human habitation, where raw milk consumption is prevalent, and where occupational exposure is unregulated. In South Asia, where about 57% of total milk production is correlated with buffaloes and raw milk consumption is widespread, the zoonotic risk from buffalo TB is substantial [199,200]. Similarly, in sub-Saharan Africa, where abattoir workers and pastoral communities have direct contact with potentially infected animals without adequate personal protective equipment, occupational exposure represents a significant and largely unquantified risk [201]. Addressing this complex zoonotic threat requires a One Health approach. The One Health argument is supported by evidence already presented: wildlife-livestock contact at shared water sources and aggregation points; human exposure through unpasteurised buffalo dairy products and occupational contact; and socio-economic and structural determinants beyond national income, including veterinary capacity, knowledge/attitudes/practices, and the small number of countries with integrated zoonotic-TB surveillance. This framework recognizes that human, animal, and environmental health are inextricably linked and that effective disease control requires coordinated action across these three domains. However, a systematic review found that only 4 of 119 WHO signatory countries (3.4%) had implemented integrated One Health surveillance for zoonotic TB, all of which were high-income countries [23]. This represents a critical gap: the countries with the highest buffalo TB burden and the greatest zoonotic risk are precisely those with the least capacity for integrated surveillance. Bridging this gap will require substantial investment in veterinary and public health infrastructure, cross-sectoral data sharing, and capacity building in low- and middle-income countries. Critically, the economic case for such investments should be made explicit: the costs of inaction—including human health burdens, livestock productivity losses, and trade restrictions—likely far exceed the costs of establishing and maintaining integrated surveillance systems. Future research should prioritize economic evaluations of One Health interventions to provide evidence for policy makers.

Several limitations merit consideration. First, significant publication bias suggests underrepresentation of small studies with low or zero prevalence, potentially inflating the pooled estimate. Trim-and-fill analysis confirmed robustness, but this issue remains inherent to meta-analyses of observational data. Future primary research should actively publish negative or low-prevalence field surveys to mitigate this reporting bias in subsequent global syntheses. Second, although the search was global in scope, the evidence base does not provide complete global coverage: the 104 studies originated from only 17 countries, and several major buffalo-producing regions—including the Middle East, Central Asia, large parts of Southeast Asia, much of sub-Saharan Africa, and parts of South America—contributed few or no published prevalence studies. The pooled estimate is therefore more representative of research-active regions than of the true global distribution of buffalo TB, and the observed geographic patterns may partly reflect the availability of published data rather than genuine differences in disease burden. This limits generalizability and should guide the geographic prioritisation of future primary surveys. This geographic gap should inform future primary research priorities: there is an urgent need for surveillance studies in underrepresented regions to refine global burden estimates and inform regional control strategies. The restriction to English- and Chinese-language reports, the omission of Scopus, and the absence of a dedicated grey-literature search may have excluded some eligible studies and are acknowledged as limitations. Third, all studies were cross-sectional, precluding causal inference and the ability to distinguish incident from prevalent infections or active from latent disease. Consequently, the risk factors identified in this study should be interpreted as risk indicators rather than causal determinants, and future longitudinal studies are needed to establish temporal relationships and investigate disease progression pathways. Fourth, heterogeneity arising from non-standardized diagnostic methods and test cutoffs could not be fully resolved through subgroup analyses. This limitation underscores the need for standardized reporting of diagnostic protocols and harmonized test interpretation criteria to improve the comparability of future studies. Fifth, several subgroup analyses had small sample sizes, yielding wide confidence intervals. These results should therefore be interpreted with appropriate caution, and the small-sample subgroups should be prioritized for replication in larger studies. Sixth, meta-regression was limited to univariate (single-covariate) analyses because many covariates were incompletely reported across studies and several are strongly collinear (e.g., region, income level and climate); consequently the combined contribution of covariates to heterogeneity was not jointly modelled, the reported subgroup differences are exploratory, and substantial residual heterogeneity remains unexplained. Multivariable meta-regression on more completely reported data is a priority for future updates. Future meta-analyses with larger sample sizes and more comprehensive data extraction could benefit from multivariate meta-regression to better disentangle the effects of interrelated risk factors. Seventh, the review was not prospectively registered in PROSPERO or another public registry; future reviews in this field should be registered prospectively to enhance transparency and reduce the risk of selective reporting. This limitation should be acknowledged transparently, and future reviews in this field should be registered prospectively to enhance methodological transparency and reduce the risk of selective reporting. Ninth, study-level quality was assessed with an author-developed reporting-quality score rather than an externally validated risk-of-bias instrument for prevalence studies (e.g., the Hoy et al. tool or the Joanna Briggs Institute checklist); the score therefore reflects reporting completeness rather than formal risk of bias, and the quality-related findings should be interpreted with this caveat (study quality did not significantly affect the pooled estimate, *p* = 0.719).

## 5. Conclusions

This systematic review and meta-analysis presents the first global synthesis of buffalo tuberculosis epidemiology, confirming that buffaloes are disproportionately affected hosts and an underrecognized reservoir of *M. bovis* infection. The 7.4% global pooled prevalence establishes a critical epidemiological baseline and unequivocally demonstrates that buffaloes must be explicitly included in national and regional bTB control strategies. The disease is driven by multidimensional risk factors, including advanced age, poor body condition, and draught use at the host level, as well as free-ranging management, large herd size, and tropical savanna climate at the management and environmental levels, and limited veterinary infrastructure and economic constraints at the socioeconomic level. These findings collectively point to a clear set of actionable priorities: at the host level, surveillance should prioritize older animals (particularly those ≥15 years), draught buffaloes, and animals with poor body condition; at the management level, interventions should target large herds, free-ranging systems, and high-risk ecological zones (e.g., the 20–30° S latitude band and tropical savanna climates); at the policy level, strengthening veterinary infrastructure and addressing socioeconomic disparities in low-income countries must be central to any sustainable control strategy. Although global prevalence has declined over time, significant disparities persist, with high-burden regions in sub-Saharan Africa and parts of South America remaining hotspots. This uneven progress demands geographically differentiated control approaches: sustained investment and intensified surveillance in high-burden regions; maintenance and consolidation of gains in lower-prevalence areas; and international support, including technology transfer and capacity building, to bridge the structural inequities that perpetuate high burden in low-income countries. From a public health perspective, the zoonotic risk—through unpasteurized milk, occupational exposure, and detection of both *M. bovis* and *M. tuberculosis* in buffaloes—highlights an urgent need to integrate buffalo TB into One Health surveillance, which remains limited in most countries. Pasteurisation should not be assumed to be routine in the high-burden regions of concern, especially for buffalo milk: in major buffalo-dairy settings a large share of milk is produced by smallholders and marketed or consumed raw, and buffaloes supply a substantial fraction of the milk (for example in Pakistan) [202]. Pasteurisation is therefore an important but context-dependent food-safety objective rather than a universally implemented safeguard, which heightens the zoonotic relevance of *M. bovis* in buffalo milk. The economic case for this integration is compelling: investing in One Health surveillance now is likely to yield substantial long-term returns by preventing human infections, reducing livestock productivity losses, and avoiding trade restrictions. To address these challenges, key control recommendations include targeted surveillance of high-risk buffalo groups using CITT or IGRA-based testing, universal pasteurization of buffalo milk in endemic areas, enhanced abattoir surveillance with mandatory ante-mortem and post-mortem inspection, inclusion of buffalo TB in national bTB control programs with dedicated support for low-income countries, and establishment of standardized global surveillance networks for real-time monitoring. Critically, these technical recommendations must be underpinned by parallel investments in community awareness and behavior change—particularly regarding raw milk consumption, reporting of sick animals, and occupational hygiene—to ensure sustainable impact. Future research should prioritize longitudinal cohort studies to establish causal relationships, whole-genome sequencing of transmission networks to trace cross-species transmission, economic evaluations of One Health interventions to guide policy decisions, and operational research on the feasibility and cost-effectiveness of tiered diagnostic algorithms in different epidemiological and resource contexts. Ultimately, this study aims to provide the epidemiological foundation for these efforts and for developing context-specific strategies to control buffalo TB globally. The evidence presented here leaves no doubt that buffalo TB is a significant and underrecognized threat to animal health, public health, and rural livelihoods. Immediate policy integration is warranted: we call on governments, international organizations, and the research community to prioritize buffalo TB in global health and agricultural agendas, and to translate the evidence from this meta-analysis into coordinated, adequately resourced, and context-adapted control programs.

## Figures and Tables

**Figure 3:**
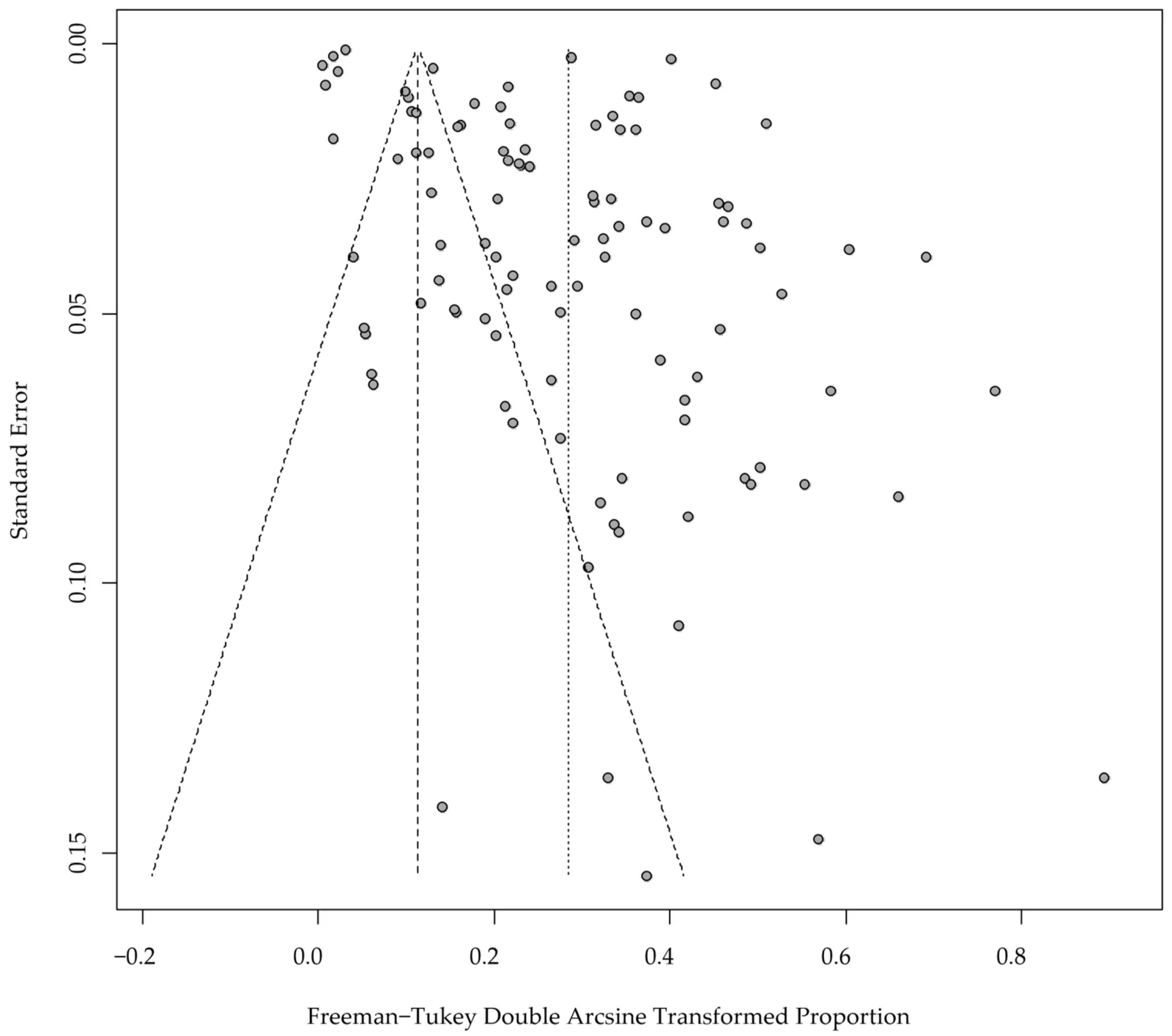
Funnel plot with pseudo 95% confidence intervals for global buffalo TB studies. Each dot represents an individual study. The vertical line indicates the pooled estimate. The diagonal dashed lines represent the pseudo 95% confidence intervals, within which 95% of studies would be expected to fall in the absence of publication bias.

**Figure 4:**
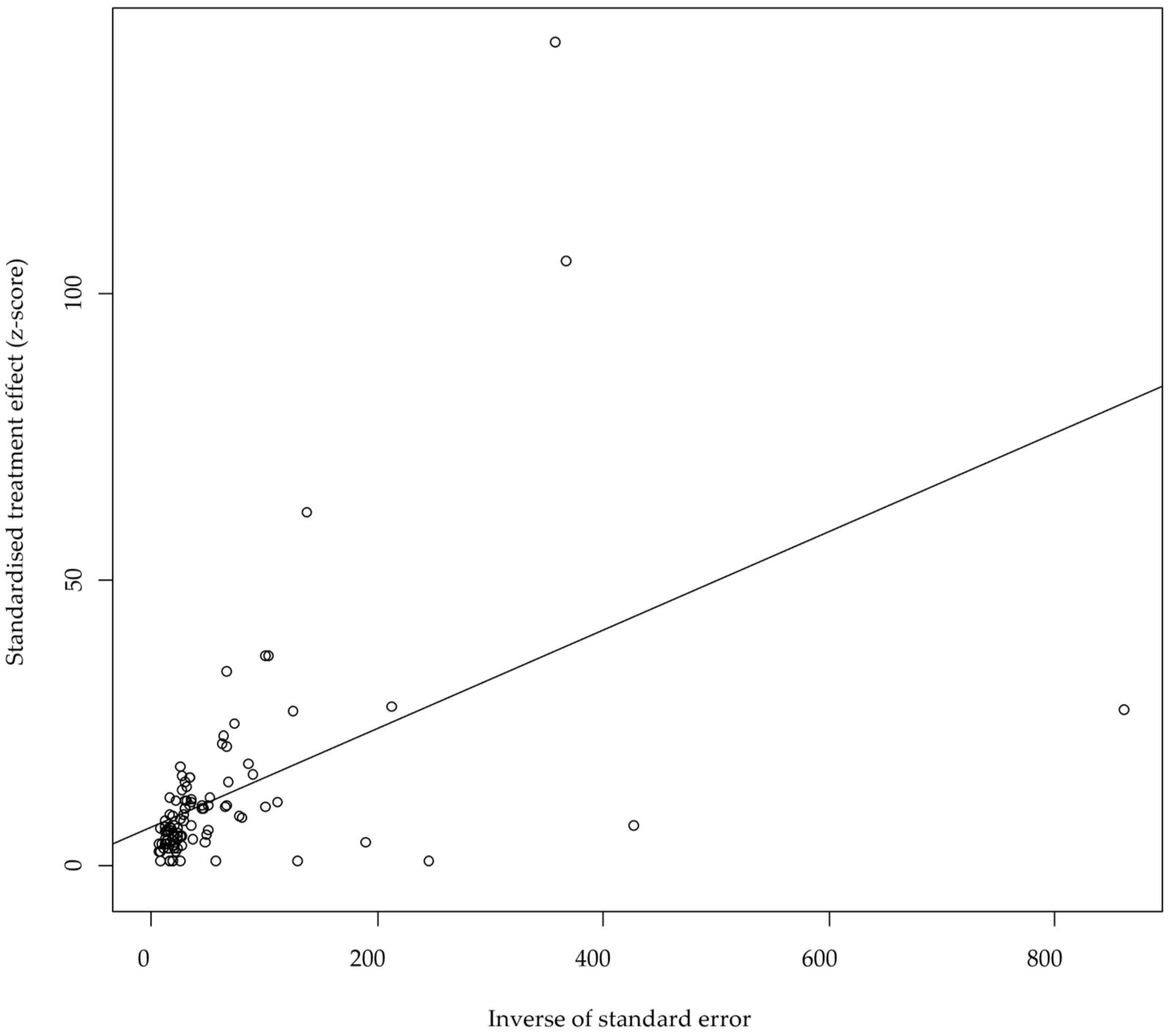
Egger’s regression test for funnel plot asymmetry. Each dot represents an individual study included in the meta-analysis. The solid line indicates the regression slope.

**Figure 5:**
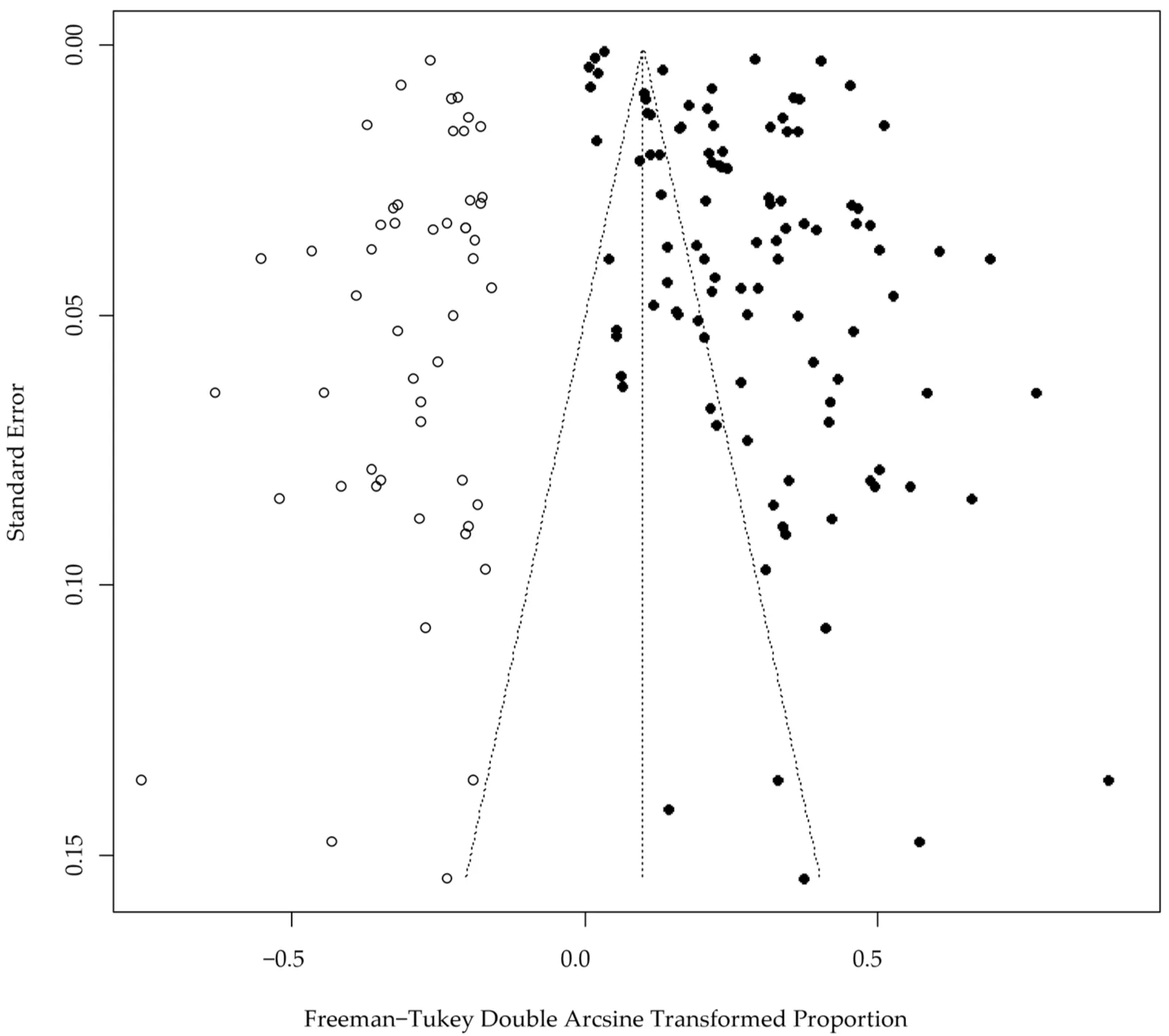
Adjusted funnel plot after trim-and-fill analysis for publication bias correction. Each dot represents an individual study. The vertical line indicates the adjusted pooled estimate. The diagonal dashed lines represent the pseudo 95% confidence interval. Imputed studies (filled circles) are shown to correct for funnel plot asymmetry.

**Table 1:** Normality test results for raw and transformed prevalence estimates.

Conversion Form	W	*p*
PRAW	0.816	4.509 × 10^−10^
PLN	NaN	NA
PLOGIT	NaN	NA
PAS	0.970	0.018
PFT	0.958	0.002

PRAW: original rate; PLN: logarithmic conversion; PLOGIT: logit transformation; PAS: arcsine transformation; PFT: double-arcsine transformation; NaN: meaningless number; NA: missing data.

**Table 2:** Included studies of tuberculosis in buffaloes wordwide.

References	Country	Sampling Time	Detection Method *	No. Tested	No. Positive	Prevalence	Quality Score
**Africa**							
Guilbride et al. (1963) [38]	Uganda	1961.9 and 1962.1	PM *	13	8	61.5%	4
Elmossalami et al. (1976) [39]	Egypt	UN	Culture	12	0	0.0%	3
Woodford (1982) [40]	Uganda	UN	PM	116	29	25.0%	3
Bengis et al. (1996) [41]	South Africa	1990.7	PM	57	9	15.8%	5
Rodwell (2000) [42]	South Africa	1991–1992 and 1998	PM	2687	322	12.0%	4
De Vos et al. (2001) [43]	South Africa	1991–1992	PM	1122	266	23.7%	5
Grobler et al. (2002) [44]	South Africa	2000	IFN-γ-ELISA *	608	9	1.5%	4
Cleaveland et al. (2005) [45]	Tanzania	1998–2001	TB ELISA *	107	1	0.9%	5
Kalema–Zikusoka et al. (2005) [46]	Uganda	1997.5–1997.6	IFN-γ-ELISA	38	8	21.1%	5
Jolles et al. (2008) [47]	South Africa	2001–2006	IFN-γ-ELISA	1362	147	10.8%	4
De Garine–Wichatitsky et al. (2010) [48]	Zimbabwe	2008.10.9–2008.10.13	IFN-γ-ELISA	38	4	10.5%	3
Parsons et al. (2011) [49]	South Africa	2010	TST *	174	40	23.0%	5
Munang’andu et al. (2011) [50]	Zambia	UN	TST	86	0	0.0%	3
Parsons et al. (2012) [51]	South Africa	2011.6	TST	217	24	11.1%	4
El–Seedy et al. (2013) [52]	Egypt	UN	TST	2550	26	1.0%	3
Clifford et al. (2013) [53]	Tanzania	2011	IFN-γ-ELISA	30	3	10.0%	4
Roug (2013) [54]	South Africa	UN	TST	229	30	13.1%	3
Jori et al. (2013) [55]	Botswana	2010.10	IFN-γ-ELISA	130	2	1.5%	4
Caron et al. (2013) [56]	Zimbabwe	2009.11	IFN-γ-ELISA	10	1	10.0%	5
Zahran et al. (2014) [57]	Egypt	UN	TST	480	27	5.6%	3
Goosen et al. (2014) [58]	South Africa	2013	TST	171	55	32.2%	5
Tanner et al. (2015) [59]	Mozambique	UN	IFN-γ-ELISA	62	0	0.0%	3
Roex et al. (2016) [60]	South Africa	2000	TST	4681	890	19.0%	4
Van der Heijden et al. (2016) [61]	South Africa	2013.6	TST	35	13	37.1%	4
Hlokwe et al. (2016) [62]	South Africa	2013.6	TST	51	8	15.7%	5
Abdel–Moein et al. (2016) [63]	Egypt	UN	PCR *	34	3	8.8%	3
Katale et al. (2017) [64]	Tanzania	2010–2013	PCR	55	2	3.6%	4
Katale et al. (2017) [65]	Tanzania	2012.4.5–2012.4.11	IFN-γ-ELISA	102	2	2.0%	5
Clarke et al. (2018) [66]	South Africa	UN	TST	286	55	19.2%	3
Bernitz et al. (2018) [67]	South Africa	2016.7	TST	226	49	21.7%	5
Elsayed and Amer (2019) [68]	Egypt	2011–2016	TST	530	24	4.5%	4
Hlokwe and Mogano (2020) [69]	South Africa	2017–2018	PCR	65	11	16.9%	5
Van der Heijden et al. (2020) [70]	South Africa	2015–2017	TST	997	124	12.4%	4
Roug et al. (2020) [71]	Tanzania	2014.10–2017.9	IFN-γ-ELISA	40	9	22.5%	4
Khalifa et al. (2021) [72]	Egypt	UN	PCR	100	7	7.0%	3
Woodburn et al. (2021) [73]	South Africa	2001–2015	PM	275	55	20.0%	3
Elsayed et al. (2022) [74]	Egypt	2016–2019	TST	1500	18	1.2%	4
Borham et al. (2022) [75]	Egypt	2018.7–2019.1	PM	181	6	3.3%	4
Mahmoud et al. (2023) [76]	Egypt	2020.10–2021.9	PM	178	3	1.7%	4
**Asia**							
Wang et al. (1980) [77]	China	1976–1978	TST	32	5	15.6%	3
Nigam et al. (1980) [78]	India	UN	Radiodiagnosis	217	24	11.1%	3
Sun and Cao (1981) [79]	China	1980–1981	TST	11	3	27.3%	3
Pei and Zhang (1982) [80]	China	UN	TST	158	64	40.5%	2
Li et al. (1989) [81]	China	1988.8.22–1988.8.26	TST	37	10	27.0%	4
Wei et al. (2001) [82]	China	1999.11	TST	90	0	0.0%	4
Fang et al. (2003) [83]	China	2002.11–2003.1	TST	46	3	6.5%	4
Pun et al. (2004) [84]	Nepal	2003.9	TST	654	35	5.4%	3
Li (2005) [85]	China	1999 and 2001–2002	TST	13	1	7.7%	4
Khan and Khan (2007) [86]	Pakistan	UN	TST	2526	321	12.7%	2
Khan et al. (2008) [87]	Pakistan	UN	TST	159	16	10.1%	4
Mumtaz et al. (2008) [88]	Pakistan	UN	TST	31	3	9.7%	3
Xu (2009) [89]	China	UN	TST	60	29	48.3%	3
Chaudhry et al. (2009) [90]	Pakistan	2007.5–2008.4	PCR	500	25	5.0%	4
Xie et al. (2010) [91]	China	UN	TST	1083	104	9.6%	3
Xie et al. (2010) [92]	China	UN	TST	1850	78	4.2%	4
Javed et al. (2010) [93]	Pakistan	2007.5–2007.7	TST	1092	28	2.6%	4
Wang et al. (2012) [94]	China	2011	TST	96	3	3.1%	4
Arshad et al. (2012) [95]	Pakistan	2005–2008	TST	1052	26	2.5%	5
Javed et al. (2012) [96]	Pakistan	UN	TST	965	109	11.3%	2
Khan et al. (2012) [97]	Pakistan	UN	TST	100	2	2.0%	3
Vongxay et al. (2012) [98]	Lao PDR	2006.4–2006.5	TB ELISA	604	7	1.2%	4
Li et al. (2013) [99]	China	UN	TST	304	32	10.5%	3
Xu et al. (2013) [100]	China	2010–2011	TST	1580	17	1.1%	4
Lao et al. (2015) [101]	China	2012–2014	TST	9000	4	0.0%	3
Basit et al. (2015) [102]	Pakistan	UN	PCR	50	2	4.0%	4
Akhtar et al. (2015) [103]	Pakistan	UN	TST	37	8	21.6%	3
Duan (2016) [104]	China	2013	TST	304	12	3.9%	4
Singh et al. (2016) [105]	India	UN	PCR	26	2	7.7%	2
Khattak et al. (2016) [106]	Pakistan	2014.6	TST	188	15	8.0%	5
Barua et al. (2017) [107]	India	UN	TST	72	10	13.9%	3
Dabade et al. (2017) [108]	India	UN	TST	99	12	12.1%	3
Wang (2018) [109]	China	UN	TST	85	3	3.5%	3
Basit et al. (2018) [110]	Pakistan	UN	PCR	64	4	6.3%	4
Aslam et al. (2019) [111]	Pakistan	UN	TST	191	19	9.9%	3
Malhi et al. (2020) [112]	Pakistan	2014–2015	TST	120	5	4.2%	5
Sidhu et al. (2020) [113]	India	UN	TST	134	6	4.5%	4
Brahma et al. (2020) [114]	India	UN	PCR	160	0	0.0%	4
He et al. (2021) [115]	China	2018–2020	TST	311	29	9.3%	4
Li et al. (2021) [116]	China	2018–2020	TST	4178	0	0.0%	3
Teng et al. (2021) [117]	China	2015–2020	TST	15,038	0	0.0%	3
Rehman et al. (2021) [118]	Pakistan	UN	TST	627	27	4.3%	3
Joseph et al. (2021) [119]	India	UN	TST	123	8	6.5%	3
Singhla and Boonyayatra (2022) [120]	Thailand	2020.4–2021.3	PM	89	17	19.1%	5
Zhao et al. (2024) [121]	China	2018–2023	TST	45,597	12	0.0%	3
Maqsood et al. (2024) [122]	Pakistan	2021.11–2022.3	PM	3140	30	1.0%	4
Kumar et al. (2024) [2]	India	UN	TST	543	4	0.7%	3
**Europe**							
Schiavo et al. (2013) [123]	Italy	UN	TST	66	0	0.0%	3
Peruzy et al. (2024) [124]	Italy	2018–2022	PM	185,583	186	0.1%	3
Martucciello et al. (2024) [125]	Italy	2017–2020	IFN-γ-ELISA	32,040	4895	15.3%	4
**Oceania**							
McCool and Newton-Tabrett (1979) [126]	Australia	1966–1974	PM	33,755	2714	8.0%	3
Hein and Tomasovic (1981) [127]	Australia	1979.4–1979.10	PM	11,322	193	1.7%	4
Wood et al. (1991) [128]	Australia	UN	TST	490	25	5.1%	3
**South America**							
Ribeiro et al. (2012) [129]	Brazil	2006–2008	PM	123	10	8.1%	3
Zarden et al. (2013) [130]	Brazil	2011	TST	21	3	14.3%	4
Barbosa et al. (2014) [131]	Brazil	2009–2011	TST	3917	180	4.6%	4
Albernaz et al. (2015) [132]	Brazil	UN	TST	1140	53	4.6%	3
Minharro et al. (2016) [133]	Brazil	UN	TST	212	31	14.6%	3
Carneiro et al. (2019) [134]	Brazil	2016.7–2018.2	PM	229	45	19.7%	4
Carneiro et al. (2020) [135]	Brazil	2016.7–2017.2	Culture	287	27	9.4%	4
Santos et al. (2021) [136]	Brazil	2016.1–2018.10	PM	798	0	0.0%	4
Carneiro et al. (2022) [137]	Brazil	2019.2–2019.8	SMS *	159	6	3.8%	4
Silva et al. (2023) [138]	Brazil	2016.1–2018.12	PM	2016	62	3.1%	4
Teixeira et al. (2024) [139]	Brazil	2019.12.2–2020.4.1	PM	327	5	1.5%	4
Oliveira et al. (2024) [140]	Brazil	2018–2020	TST	60	18	30.0%	5

Sampling time written explicitly (e.g., “1961.9”; “UN” indicates that sampling time was not reported); prevalence rounded to one decimal place; a Country column added, so columns are reference, country, sampling period, method, tested, positive, prevalence, and reporting-quality score. PM *: Post mortem; PCR *: Polymerase chain reaction; TST *: Tuberculin skin test; IFN-γ-ELISA *: Interferon-γ-enzyme linked immunosorbent assay; TB ELISA *: bTB enzyme linked immunosorbent assay; SMS *: Shotgun metagenomic sequencing.

**Table 3:** Pooled prevalence of tuberculosis in buffaloes wordwide.

		No. Studies	No. Tested	No. Positive	% (95% CI *)
Publication years					
	Before 2000	12	46,220	3084	15.8% (7.7–26.0)
	2000–2010	15	10,003	1186	9.3% (4.6–15.3)
	2010–2020	49	34,727	2077	7.3% (5.2–9.6)
	2020–2025	28	294,666	5570	4.5% (2.2–7.4)
Sampling years					
	Before 2000	15	50,020	3645	12.8% (6.2–20.9)
	2000–2010	24	10,086	1095	6.8% (3.2–11.3)
	2010–2020	49	69,330	3579	5.4% (3.1–8.2)
	2020–2025	11	50,543	1996	1.5% (0.0–4.9)
Season					
	Dry season	12	12,351	341	11.8% (4.5–21.8)
	Rainy season	8	2085	92	4.0% (0.3–10.1)
Longitude					
	0–30° E	9	218,371	5191	7.9% (2.1–16.7)
	30–60° E	43	12,918	1963	10.0% (6.5–14.1)
	30–60° W	5	2755	134	8.4% (2.1–18.2)
	60–90° E	17	7524	185	3.5% (2.0–5.3)
	90–120° E	20	32,790	241	4.8% (1.6–9.2)
Latitude					
	0–20° S	21	2944	102	3.0% (0.0–8.8)
	0–30° N	21	33,436	263	4.2% (1.5–8.0)
	20–30° S	27	12,426	2040	16.0% (12.4–20.0)
	30–60° N	25	225,552	5309	4.3% (2.5–6.5)
Climate					
	Mediterranean climate	10	220,592	5162	3.2% (0.9–6.5)
	Tropical savanna climate	54	59,318	5045	10.1% (7.2–13.6)
	Tropical monsoon climate	12	5932	551	7.5% (3.8–12.3)
	Tropical desert climate	15	6611	167	3.8% (2.3–5.7)
	Tropical rainforest climate	12	9268	437	6.8% (3.1–11.6)
	Temperate continental climate	1	188	15	8.0% (4.5–12.3)
	Subtropical monsoon climate	19	32,701	224	4.3% (1.3–8.6)
Region *					
	Africa	39	19,635	2290	10.1% (6.9–13.7)
	Asia	47	93,436	1174	6.0% (3.9–8.6)
	Europe	3	217,689	5081	2.6% (0.0–15.3)
	Oceania	3	45,567	2932	4.6% (1.5–9.1)
	South America	12	9289	440	7.1% (3.2–12.4)
Subregion *					
	East Asia	20	79,873	409	6.4% (2.2–12.4)
	Eastern Africa	8	501	62	13.5% (3.9–27.1)
	North Africa	9	5565	114	2.7% (1.2–4.6)
	Oceania	3	45,567	2932	4.6% (1.5–9.1)
	South America	12	9289	440	7.1% (3.2–12.4)
	South Asia	25	12,870	741	5.7% (3.9–7.9)
	Southeast Asia	2	693	24	7.4% (0.0–33.5)
	Southern Africa	22	13,569	2114	13.0% (8.8–17.8)
	Southern Europe	3	217,689	5081	2.6% (0.0–15.3)
Development status					
	Developed country	6	263,256	8013	3.6% (0.5–9.1)
	Developing country	89	120,671	3810	7.6% (5.8–9.6)
	Landlocked developing country	9	1689	94	9.7% (1.8–21.8)
Income level					
	High-income economy	6	263,256	8013	3.6% (0.5–9.1)
	Upper-middle-income economy	51	102,624	2975	9.7% (6.8–13.1)
	Lower-middle-income economy	42	19,421	884	4.8% (3.5–6.4)
	Low-income economy	5	315	45	13.7% (0.0–40.6)
BCS *					
	Good	7	1143	37	4.5% (0.4–11.3)
	Poor	2	227	18	33.3% (0.1–99.8)
Gender					
	Female	22	16,501	671	5.7% (3.3–8.5)
	Male	11	6621	137	3.5% (0.0–12.6)
Age					
	<1	5	51	0	0.0% (0.0–0.4)
	1–5	14	1011	32	0.5% (0.0–3.7)
	5–15	14	2622	265	11.7% (4.7–21.0)
	15–20	3	30	9	34.2% (2.6–75.4)
Weight (kg)					
	≤500	6	1694	66	3.4% (1.2–6.6)
	>500	5	988	98	9.9% (4.0–17.8)
Milk Yield (Liters)					
	<5	5	648	14	1.5% (0.5–2.8)
	5–10	5	1489	76	4.1% (2.2–6.6)
	10–15	3	690	69	7.3% (0.0–30.2)
	>15	2	50	4	9.3% (0.0–59.8)
Parity					
	<5	5	1740	81	4.6% (2.4–7.5)
	5–10	2	348	34	11.0% (0.5–31.3)
	>10	1	44	3	6.8% (0.9–16.6)
Herd size					
	<100	6	1087	32	0.0% (0.0–0.0)
	100–1000	6	678	81	10.3% (4.8–17.1)
	>1000	1	406	40	9.9% (7.1–13.0)
Purpose					
	Dairy buffalo	32	88,432	946	4.1% (2.6–5.9)
	Draught buffalo	4	219	78	28.0% (15.3–42.6)
	Wild buffalo	29	13,795	2121	12.7% (8.6–17.4)
Source					
	Abattoir	15	238,467	3315	3.5% (1.3–6.7)
	Farm	31	63,044	1100	5.9% (4.0–8.1)
	Free-ranging	30	13,875	2121	12.0% (8.0–16.7)
	Rural/Village	7	2394	103	6.3% (2.3–11.8)
Detection method *					
	Culture	8	4443	105	5.7% (1.5–12.1)
	IFN-γ-ELISA	23	37,501	5248	9.1% (5.0–14.2)
	PCR	25	11,979	249	6.8% (3.2–11.5)
	PM	18	242,011	3960	8.3% (3.8–14.2)
	Radiodiagnosis	1	217	24	11.1% (7.2–15.6)
	SMS	1	159	6	3.8% (1.3–7.4)
	TB ELISA	9	2131	193	6.2% (2.3–11.5)
	TST	62	107,791	2811	7.8% (5.6–10.3)
	ZN	4	1430	200	10.1% (5.5–15.7)
Tuberculin test *					
	SITT	30	84,968	596	6.1% (3.1–10.0)
	CITT	30	21,737	2160	9.9% (6.8–13.5)
Sample					
	Bacterial colonies	4	514	48	9.1% (6.7–11.9)
	Blood	25	37,761	5302	9.8% (5.1–15.6)
	Fecal	1	26	2	7.7% (0.2–21.8)
	Milk	8	669	48	8.6% (2.8–16.6)
	Serum	9	2131	193	6.2% (2.3–11.5)
	TB–like lesions	6	12,881	58	1.3% (0.0–5.0)
	Tissue (Lymph nodes)	17	3661	350	7.1% (3.9–11.0)
Species *					
	*M. bovis*	17	5366	193	7.4% (3.0–13.3)
	*M. orygis*	2	3205	16	3.3% (0.0–20.7)
	*M. tuberculosis*	5	3377	29	3.5% (0.0–12.0)
	MTBC	1	31	11	35.5% (19.4–53.3)
Quality level					
	2–3	44	307,394	4095	7.0% (4.5–10.0)
	4–5	60	78,222	7822	7.6% (5.5–10.0)
Total		104	385,616	11,917	7.4% (5.7–9.2)

CI *: Confidence interval. BCS *: Body condition score. Region *: Oceania: Australia; Africa: Botswana, Egypt, Mozambique, South Africa, Tanzania, Uganda, Zambia, Zimbabwe; South America: Brazil; Asia: China, India, Lao PDR, Nepal, Pakistan, Thailand; Europe: Italy. Subregion *: East Asia: China; South Asia: Pakistan, India, Nepal; Southeast Asia: Lao PDR, Thailand; Eastern Africa: Tanzania, Uganda; North Africa: Egypt; Southern Africa: South Africa, Mozambique, Zambia, Botswana, Zimbabwe; Oceania: Australia; South America: Brazil; Southern Europe: Italy. Detection method *: IFN-γ-ELISA: Interferon-γ-enzyme linked immunosorbent assay; PCR: Polymerase chain reaction; PM: Post mortem; SMS: Shotgun metagenomic sequencing; TB ELISA: bTB enzyme linked immunosorbent assay; TST: Tuberculin skin test; ZN: Ziehl-Neelsen. Tuberculin test *: SITT: Single intradermal tuberculin test; CITT: Comparative intradermal tuberculin test. Species *: *M. bovis*: *Mycobacterium bovis*; *M. orygis*: *Mycobacterium orygis*; *M. tuberculosis*: *Mycobacterium tuberculosis*; MTBC: *Mycobacterium tuberculosis* complex.

**Table 4:** Pooled prevalence of buffalo tuberculosis by country.

Country	No. Studies	Region	No. Tested	No. Positive	Prevalence (%)	% (95% CI)
Australia	3	Oceania	45,567	2932	4.6%	1.5–9.1
Botswana	1	Africa	130	2	1.5%	0.0–4.6
Brazil	12	South America	9289	440	7.1%	3.2–12.4
China	20	Asia	79,873	409	6.4%	2.2–12.4
Egypt	9	Africa	5565	114	2.7%	1.2–4.6
India	8	Asia	1374	66	5.4%	1.8–10.6
Italy	3	Europe	217,689	5081	2.6%	0.0–15.3
Lao PDR	1	Asia	604	7	1.2%	0.4–2.2
Mozambique	1	Africa	62	0	0.0%	0.0–2.8
Nepal	1	Asia	654	35	5.4%	3.8–7.2
Pakistan	16	Asia	10,842	640	5.9%	3.8–8.5
South Africa	17	Africa	13,243	2107	16.6%	12.6–21.0
Tanzania	5	Africa	334	17	5.6%	0.7–13.9
Thailand	1	Asia	89	17	19.1%	11.5–28.0
Uganda	3	Africa	167	45	32.0%	12.9–54.6
Zambia	1	Africa	86	0	0.0%	0.0–2.0
Zimbabwe	2	Africa	48	5	9.7%	2.1–20.6

## Data Availability

The original contributions presented in the study are included in the article/Supplementary Materials; further inquiries can be directed to the corresponding author.

## Supplementary Materials

The following supporting information can be downloaded at https://www.mdpi.com/article/10.3390/ani16172774/s1. Table S1. PRISMA checklist item. Table S2. The specific search strategy. Table S3. The code in R for this meta–analysis. Table S4. Included studies and quality scores. Table S5. Egger’s for publication bias. Table S6. Pooled prevalence of tuberculosis in buffalos wordwide. Figure S1. Funnel plot with pseudo 95% confidence intervals for examining publication bias in the publication years subgroup of global buffalo tuberculosis. Figure S2. Funnel plot with pseudo 95% confidence intervals for examining publication bias in the sampling years subgroup of global buffalo tuberculosis. Figure S3. Funnel plot with pseudo 95% confidence intervals for examining publication bias in the season subgroup of global buffalo tuberculosis. Figure S4. Funnel plot with pseudo 95% confidence intervals for examining publication bias in the longitude subgroup of global buffalo tuberculosis. Figure S5. Funnel plot with pseudo 95% confidence intervals for examining publication bias in the latitude subgroup of global buffalo tuberculosis. Figure S6. Funnel plot with pseudo 95% confidence intervals for examining publication bias in the climate subgroup of global buffalo tuberculosis. Figure S7. Funnel plot with pseudo 95% confidence intervals for examining publication bias in the region subgroup of global buffalo tuberculosis. Figure S8. Funnel plot with pseudo 95% confidence intervals for examining publication bias in the subregion subgroup of global buffalo tuberculosis. Figure S9. Funnel plot with pseudo 95% confidence intervals for examining publication bias in the development status subgroup of global buffalo tuberculosis. Figure S10. Funnel plot with pseudo 95% confidence intervals for examining publication bias in the income level subgroup of global buffalo tuberculosis. Figure S11. Funnel plot with pseudo 95% confidence intervals for examining publication bias in the BCS subgroup of global buffalo tuberculosis. Figure S12. Funnel plot with pseudo 95% confidence intervals for examining publication bias in the gender subgroup of global buffalo tuberculosis. Figure S13. Funnel plot with pseudo 95% confidence intervals for examining publication bias in the age subgroup of global buffalo tuberculosis. Figure S14. Funnel plot with pseudo 95% confidence intervals for examining publication bias in the weight (kg) subgroup of global buffalo tuberculosis. Figure S15. Funnel plot with pseudo 95% confidence intervals for examining publication bias in the milk yield (Liters) subgroup of global buffalo tuberculosis. Figure S16. Funnel plot with pseudo 95% confidence intervals for examining publication bias in the parity subgroup of global buffalo tuberculosis. Figure S17. Funnel plot with pseudo 95% confidence intervals for examining publication bias in the herd size subgroup of global buffalo tuberculosis. Figure S18. Funnel plot with pseudo 95% confidence intervals for examining publication bias in the purpose subgroup of global buffalo tuberculosis. Figure S19. Funnel plot with pseudo 95% confidence intervals for examining publication bias in the source subgroup of global buffalo tuberculosis. Figure S20. Funnel plot with pseudo 95% confidence intervals for examining publication bias in the detection method subgroup of global buffalo tuberculosis. Figure S21. Funnel plot with pseudo 95% confidence intervals for examining publication bias in the tuberculin test subgroup of global buffalo tuberculosis. Figure S22. Funnel plot with pseudo 95% confidence intervals for examining publication bias in the sample subgroup of global buffalo tuberculosis. Figure S23. Funnel plot with pseudo 95% confidence intervals for examining publication bias in the species subgroup of global buffalo tuberculosis. Figure S24. Funnel plot with pseudo 95% confidence intervals for examining publication bias in the quality level subgroup of global buffalo tuberculosis.
