# Supplementary material for: The Global Prevalence of Tuberculosis Infection in Buffaloes: A Systematic Review and Meta-Analysis
Source: Animals (Basel). 2026 Sep 3;16(17):2774. doi: 10.3390/ani16172774 (PMC13565276; doi:10.3390/ani16172774)
Supplement: Supplementary file 1 [file animals-16-02774-s001.zip › animals-4448897-supplementary.pdf]

Table S1. PRISMA checklist item.

Section/topic	#	Checklist item	Reported on page #
**TITLE**			
Title	1	The global prevalence of tuberculosis infection in buffaloes: A systematic review and meta–analysis	1
**ABSTRACT**			
Structured summary	2	Provide a structured summary including, as applicable: background; objectives; data sources; study eligibility criteria, participants, and interventions; study appraisal and synthesis methods; results; limitations; conclusions and implications of key findings; systematic review registration number.	1-2
**INTRODUCTION**			
Rationale	3	Describe the rationale for the review in the context of what is already known.	2
Objectives	4	Provide an explicit statement of questions being addressed with reference to participants, interventions, comparisons, outcomes, and study design (PICOS).	2-3
**METHODS**			
Protocol and registration	5	Indicate if a review protocol exists, if and where it can be accessed (e.g., Web address), and, if available, provide registration information including registration number.	3
Eligibility criteria	6	Specify study characteristics (e.g., PICOS, length of follow-up) and report characteristics (e.g., years considered, language, publication status) used as criteria for eligibility, giving rationale.	3
Information sources	7	Describe all information sources (e.g., databases with dates of coverage, contact with study authors to identify additional studies) in the search and date last searched.	3
Search	8	Present full electronic search strategy for at least one database, including any limits used, such that it could be repeated.	3

Study selection	9	State the process for selecting studies (i.e., screening, eligibility, included in systematic review, and, if applicable, included in the meta-analysis).	3, Figure 1
Data collection process	10	Describe method of data extraction from reports (e.g., piloted forms, independently, in duplicate) and any processes for obtaining and confirming data from investigators.	3
Data items	11	List and define all variables for which data were sought (e.g., PICOS, funding sources) and any assumptions and simplifications made.	3
Risk of bias in individual studies	12	Describe methods used for assessing risk of bias of individual studies (including specification of whether this was done at the study or outcome level), and how this information is to be used in any data synthesis.	4, Figure 3, Figures S1–S24
Summary measures	13	State the principal summary measures (e.g., risk ratio, difference in means).	4
Synthesis of results	14	Describe the methods of handling data and combining results of studies, if done, including measures of consistency (e.g., I^2) for each meta-analysis.	4, Table 1
Risk of bias across studies	15	Specify any assessment of risk of bias that may affect the cumulative evidence (e.g., publication bias, selective reporting within studies).	4，Figure 4 and 5
Additional analyses	16	Describe methods of additional analyses (e.g., sensitivity or subgroup analyses, meta-regression), if done, indicating which were pre-specified.	4
**RESULTS**			
Study selection	17	Give numbers of studies screened, assessed for eligibility, and included in the review, with reasons for exclusions at each stage, ideally with a flow diagram.	4, Figure 1
Study characteristics	18	For each study, present characteristics for which data were extracted (e.g., study size, PICOS, follow-up period) and provide the citations.	5, Table 2 and 4
Risk of bias within studies	19	Present data on risk of bias of each study and, if available, any outcome level assessment (see item 12).	9
Results of individual studies	20	For all outcomes considered (benefits or harms), present, for each study: (a) simple summary data for each intervention group (b) effect estimates and confidence intervals, ideally with a forest plot.	7, Figure 2

Synthesis of results	21	Present results of each meta-analysis done, including confidence intervals and measures of consistency.	13, Table 3
Risk of bias across studies	22	Present results of any assessment of risk of bias across studies (see Item 15).	9, Figure 3, 4 and 5
Additional analysis	23	Give results of additional analyses, if done (e.g., sensitivity or subgroup analyses, meta-regression [see Item 16]).	11, 16-17, Figure 6, Table 3
**DISCUSSION**			
Summary of evidence	24	Summarize the main findings including the strength of evidence for each main outcome; consider their relevance to key groups (e.g., healthcare providers, users, and policy makers).	17-23
Limitations	25	Discuss limitations at study and outcome level (e.g., risk of bias), and at review-level (e.g., incomplete retrieval of identified research, reporting bias).	23-24
Conclusions	26	Provide a general interpretation of the results in the context of other evidence, and implications for future research.	24-25
**FUNDING**			
Funding	27	Describe sources of funding for the systematic review and other support (e.g., supply of data); role of founders for the systematic review.	26

From: Moher D, Liberati A, Tetzlaff J, Altman DG, The PRISMA Group (2009). Preferred Reporting Items for Systematic Reviews and Meta-Analyses: The PRISMA Statement. PLoS Med 6(6): e1000097. doi:10.1371/journal.pmed1000097

For more information, visit: **www.prisma-statement.org**.

Table S2. The specific search strategy

Database	Search Strategy
PubMed	((((((("Buffaloes"[Mesh]) OR (Buffalo)) OR (Bubalus)) OR (Syncerus)) OR (Water Buffaloes)) OR (Water Buffalo)) OR (Buffalo, Water)) AND (((((((("Tuberculosis"[Mesh]) OR (Tuberculoses)) OR (Kochs Disease)) OR (Koch's Disease)) OR (Koch Disease)) OR (Mycobacterium tuberculosis Infection)) OR (Infection, Mycobacterium tuberculosis)) OR (Infections, Mycobacterium tuberculosis)) OR (Mycobacterium tuberculosis Infections))
ScienceDirect	("tuberculosis") AND ("buffalo" OR "buffaloes" OR "water buffalo" OR "Bubalus") AND ("prevalence").
Web of Science	TS=("tuberculosis") AND TS= ("buffalo" OR "buffaloes" OR "water buffalo" OR "Bubalus") AND TS=("prevalence").
CNKI	(水牛 OR 非洲水牛 OR 野水牛) AND (结核 OR 结核病 OR 牛结核病 OR 分枝杆菌) AND （流行率 OR 患病率 OR 流行 OR 血清流行率）.
VIP	(水牛 OR 非洲水牛 OR 野水牛) AND (结核 OR 结核病 OR 牛结核病) AND (流行率 OR 患病率 OR 流行).
WanFang	(水牛 OR 非洲水牛 OR 野水牛) AND (结核 OR 结核病 OR 牛结核病 OR 分枝杆菌) AND (流行率 OR 患病率 OR 流行 OR 血清流行率).

Table S3. The code in R for this meta–analysis.

Logarithmic conversion (PLN)	rate<-transform [m1, log=log(event/n)];<br>shapiro.test(rate$log)
Logit transformation (PLOGIT)	rate<-transform{m1, logit=log[(event/n)/(1-event/n)]};<br>shapiro.test(rate$logit)
Arcsine transformation (PAS)	rate<-transform{m1, arcsin.size=asin[sqrt(event/(n+1))]};<br>shapiro.test(rate$arcsin)
Double-arcsine transformation (PFT)	rate<transform{m1,darcsin=0.5*[asin(sqrt(event/(n+1)))+asin((sqrt(event+1)/(n+1)))]};<br>shapiro.test(rate$darcsin)
No transformation (PRAW)	rate<-transform[m1, r= event/n];<br>shapiro.test(rate$r)
Forest plots	forest [meta1, xlim=c(-0.9, 1)]
Funnel chart	funnel (meta1)
Egger's test	metabias (meta1, method="linreg")
The sensitivity analysis	Metainf (meta1, pooled = "random") forest (metainf (meta1, pooled = "random"), xlim=c(0, 0.4))
Subgroup analysis	meta1<-metaprop(event, n, study, data=rate, sm="PLN", incr=0.5, allincr=TRUE, addincr=FALSE, title="", byvar= subgroup title, print.byvar=TRUE)
Meta-regression analysis	metareg (meta1, ~covariate title)

Table S4. Included studies and quality scores.

Reference ID	No. tested	No. positive	Prevalence (%)	Random sampling or not	Detection method clear or not	Detailed sampled method or no	Sample time clear or not	Four or more risk factors or not	Score	Study Quality
Arshad et al. (2012)	1052	26	0.02471	Y	Y	Y	Y	Y	5	High
Malhi et al. (2020)	120	5	0.04167	Y	Y	Y	Y	Y	5	High
Hlokwe and Mogano (2020)	65	11	0.16923	Y	Y	Y	Y	Y	5	High
Cleaveland et al. (2005)	107	1	0.00935	Y	Y	Y	Y	Y	5	High
Singhla and Boonyayatra (2022)	89	17	0.19101	Y	Y	Y	Y	Y	5	High
Oliveira et al. (2024)	60	18	0.30000	Y	Y	Y	Y	Y	5	High
Bengis et al. (1996)	57	9	0.15789	Y	Y	Y	Y	Y	5	High
Parsons et al. (2011)	174	40	0.22989	Y	Y	Y	Y	Y	5	High
Khattak et al. (2016)	188	15	0.07979	Y	Y	Y	Y	Y	5	High
Katale et al. (2017)	102	2	0.01961	Y	Y	Y	Y	Y	5	High
Hlokwe et al. (2016)	51	8	0.15686	Y	Y	Y	Y	Y	5	High
Goosen et al. (2014)	171	55	0.32164	Y	Y	Y	Y	Y	5	High
Caron et al. (2013)	10	1	0.10000	Y	Y	Y	Y	Y	5	High
Bernitz et al. (2018)	226	49	0.21681	Y	Y	Y	Y	Y	5	High
De Vos et al. (2001)	1122	266	0.23708	Y	Y	Y	Y	Y	5	High
Kalema-Zikusoka et al. (2005)	38	8	0.21053	Y	Y	Y	Y	Y	5	High
Fang et al. (2003)	46	3	0.06522	N	Y	Y	Y	Y	4	High
He et al. (2021)	311	29	0.09325	N	Y	Y	Y	Y	4	High
Li et al. (1989)	37	10	0.27027	N	Y	Y	Y	Y	4	High
Wang et al. (2012)	96	3	0.03125	N	Y	Y	Y	Y	4	High
Duan (2016)	304	12	0.03947	Y	Y	N	N	Y	4	High
Xu et al. (2013)	1580	17	0.01076	N	Y	Y	Y	Y	4	High
Li (2005)	13	1	0.07692	N	Y	Y	Y	Y	4	High

Wei et al. (2001)	90	0	0.00000	N	Y	Y	Y	Y	4	High
Chaudhry et al. (2009)	500	25	0.05000	Y	Y	N	Y	Y	4	High
Rodwell (2000)	2687	322	0.11984	Y	Y	N	Y	Y	4	High
Khan et al. (2008)	159	16	0.10063	N	Y	Y	Y	Y	4	High
Sidhu et al. (2020)	134	6	0.04478	Y	Y	Y	N	Y	4	High
Basit et al. (2015)	50	2	0.04000	Y	Y	Y	N	Y	4	High
Santos et al. (2021)	798	0	0.00000	N	Y	Y	Y	Y	4	High
Teixeira et al. (2024)	327	5	0.01529	N	Y	Y	Y	Y	4	High
Clifford et al. (2013)	30	3	0.10000	N	Y	Y	Y	Y	4	High
Basit et al. (2018)	64	4	0.06250	Y	Y	Y	N	Y	4	High
Elsayed et al. (2022)	1500	18	0.01200	N	Y	Y	Y	Y	4	High
Mahmoud et al. (2023)	178	3	0.01685	N	Y	Y	Y	Y	4	High
Carneiro et al. (2019)	229	45	0.19651	N	Y	Y	Y	Y	4	High
Grobler et al. (2002)	608	9	0.01480	N	Y	Y	Y	Y	4	High
Guilbride et al. (1963)	13	8	0.61538	N	Y	Y	Y	Y	4	High
Vongxay et al. (2012)	604	7	0.01159	N	Y	Y	Y	Y	4	High
Silva et al. (2023)	2016	62	0.03075	N	Y	Y	Y	Y	4	High
Maqsood et al. (2024)	3140	30	0.00955	N	Y	Y	Y	Y	4	High
Carneiro et al. (2022)	159	6	0.03774	N	Y	Y	Y	Y	4	High
Jolles et al. (2008)	1362	147	0.10793	N	Y	Y	Y	Y	4	High
Brahma et al. (2020)	160	0	0.00000	Y	Y	Y	N	Y	4	High
Borham et al. (2022)	181	6	0.03315	N	Y	Y	Y	Y	4	High
Martucciello et al. (2024)	32040	4895	0.15278	N	Y	Y	Y	Y	4	High
Roex et al. (2016)	4681	890	0.19013	N	Y	Y	Y	Y	4	High
Javed et al. (2010)	1092	28	0.02564	N	Y	Y	Y	Y	4	High
Van der Heijden et al. (2016)	35	13	0.37143	N	Y	Y	Y	Y	4	High
Zarden et al. (2013)	21	3	0.14286	N	Y	Y	Y	Y	4	High

Parsons et al. (2012)	217	24	0.11060	N	Y	Y	Y	Y	4	High
Katale et al. (2017)	55	2	0.03636	N	Y	Y	Y	Y	4	High
Jori et al. (2013)	130	2	0.01538	N	Y	Y	Y	Y	4	High
Elsayed and Amer (2019)	530	24	0.04528	N	Y	Y	Y	Y	4	High
Barbosa et al. (2014)	3917	180	0.04595	N	Y	Y	Y	Y	4	High
Van der Heijden et al. (2020)	997	124	0.12437	N	Y	Y	Y	Y	4	High
Carneiro et al. (2020)	287	27	0.09408	N	Y	Y	Y	Y	4	High
Roug et al. (2020)	40	9	0.22500	N	Y	Y	Y	Y	4	High
Hein and Tomasovic (1981)	11322	193	0.01705	N	Y	Y	Y	Y	4	High
Li et al. (2021)	4178	0	0.00000	N	Y	N	Y	Y	3	Moderate
Lao et al. (2015)	9000	4	0.00044	N	Y	N	Y	Y	3	Moderate
Li et al. (2013)	304	32	0.10526	N	Y	Y	N	Y	3	Moderate
Wang et al. (1980)	32	5	0.15625	N	Y	N	Y	Y	3	Moderate
Xu (2009)	60	29	0.48333	N	Y	Y	N	Y	3	Moderate
Wang (2018)	85	3	0.03529	N	Y	Y	N	Y	3	Moderate
Xie et al. (2010)	1083	104	0.09603	N	Y	Y	N	Y	3	Moderate
Xie et al. (2010)	1850	78	0.04216	Y	Y	Y	N	Y	3	Moderate
Teng et al. (2021)	15038	0	0.00000	N	Y	N	Y	Y	3	Moderate
Zhao et al. (2024)	45597	12	0.00026	N	Y	N	Y	Y	3	Moderate
Sun and Cao (1981)	11	3	0.27273	N	Y	N	Y	Y	3	Moderate
Elmossalami et al. (1976)	12	0	0.00000	Y	Y	N	N	Y	3	Moderate
El-Seedy et al. (2013)	2550	26	0.01020	N	Y	Y	N	Y	3	Moderate
Khan et al. (2012)	100	2	0.02000	N	Y	Y	N	Y	3	Moderate
Khalifa et al. (2021)	100	7	0.07000	N	Y	Y	N	Y	3	Moderate
Minharro et al. (2016)	212	31	0.14623	Y	Y	N	N	Y	3	Moderate
Schiavo et al. (2013)	66	0	0.00000	N	Y	Y	N	Y	3	Moderate
Barua et al. (2017)	72	10	0.13889	N	Y	Y	N	Y	3	Moderate

Rehman et al. (2021)	627	27	0.04306	N	Y	Y	N	Y	3	Moderate
Mumtaz et al. (2008)	31	3	0.09677	N	Y	Y	N	Y	3	Moderate
Ribeiro et al. (2012)	123	10	0.08130	N	Y	N	Y	Y	3	Moderate
Peruzy et al. (2024)	185583	186	0.00100	N	Y	N	Y	Y	3	Moderate
Aslam et al. (2019)	191	19	0.09948	N	Y	Y	N	Y	3	Moderate
Roug (2013)	229	30	0.13100	N	Y	Y	N	Y	3	Moderate
Woodford (1982)	116	29	0.25000	N	Y	Y	N	Y	3	Moderate
McCool and Newton-Tabrett (1979)	33755	2714	0.08040	N	Y	N	Y	Y	3	Moderate
Pun et al. (2004)	654	35	0.05352	N	Y	N	Y	Y	3	Moderate
Nigam et al. (1980)	217	24	0.11060	N	Y	Y	N	Y	3	Moderate
Woodburn et al. (2021)	275	55	0.20000	N	Y	N	Y	Y	3	Moderate
Wood et al. (1991)	490	25	0.05102	N	Y	Y	N	Y	3	Moderate
Kumar et al. (2024)	543	4	0.00737	N	Y	Y	N	Y	3	Moderate
Dabade et al. (2017)	99	12	0.12121	N	Y	Y	N	Y	3	Moderate
Zahran et al. (2014)	480	27	0.05625	N	Y	Y	N	Y	3	Moderate
Tanner et al. (2015)	62	0	0.00000	N	Y	Y	N	Y	3	Moderate
Munang'andu et al. (2011)	86	0	0.00000	N	Y	Y	N	Y	3	Moderate
De Garine-Wichatitsky et al. (2010)	38	4	0.10526	N	Y	N	Y	Y	3	Moderate
Clarke et al. (2018)	286	55	0.19231	N	Y	Y	N	Y	3	Moderate
Albernaz et al. (2015)	1140	53	0.04649	N	Y	Y	N	Y	3	Moderate
Akhtar et al. (2015)	37	8	0.21622	N	Y	Y	N	Y	3	Moderate
Abdel-Moein et al. (2016)	34	3	0.08824	N	Y	Y	N	Y	3	Moderate
Joseph et al. (2021)	123	8	0.06504	N	Y	Y	N	Y	3	Moderate
Pei and Zhang (1982)	158	64	0.40506	N	Y	N	N	Y	2	Moderate
Khan and Khan (2007)	2526	321	0.12708	N	Y	N	N	Y	2	Moderate
Javed et al. (2012)	965	109	0.11295	N	Y	N	N	Y	2	Moderate
Singh et al. (2016)	26	2	0.07692	N	Y	N	N	Y	2	Moderate

Y*: Yes; N*: No.

Table S5. Egger's for publication bias.

bias	se.bias	t	df	p-value
6.8445	1.8155	3.77	102	0.0003

Table S6. Pooled prevalence of tuberculosis in buffalos wordwide.

		No. studies	No. tested	No. positive	% (95% CI*)	Heterogeneity			Univariate meta-regression	
						χ^2	*P*-value	I^2 (%)	*P*-value	Coefficient (95% CI)
Publication years										
	Before 2000	12	46220	3084	15.8% (7.7-26.0)	1048.21	< 0.01	99.0%	0.005	0.148 (0.045 to 0.252)
	2000-2010	15	10003	1186	9.3% (4.6-15.3)	402.90	< 0.01	96.5%		
	2010-2020	49	34727	2077	7.3% (5.2-9.6)	3269.82	0	98.5%		
	2020-2025	28	294666	5570	4.5% (2.2-7.4)	17106.23	0	99.8%		
Sampling years										
	Before 2000	15	50020	3645	12.8% (6.2-20.9)	1358.99	< 0.01	99.0%	0.006	0.155 (0.043 to 0.266)
	2000-2010	24	10086	1095	6.8% (3.2-11.3)	1048.02	< 0.01	97.8%		
	2010-2020	49	69330	3579	5.4% (3.1-8.2)	10636.86	0	99.5%		
	2020-2025	11	50543	1996	1.5% (0.0-4.9)	4735.68	0	99.8%		
Season										
	Dry season	12	12351	341	11.8% (4.5-21.8)	317.31	< 0.01	96.5%	0.438	0.073 (-0.112 to 0.259)
	Rainy season	8	2085	92	4.0% (0.3-10.1)	39.33	< 0.01	82.2%		
Longitude										
	0-30°E	9	218371	5191	7.9% (2.1-16.7)	15271.80	0	99.9%		
	30-60°E	43	12918	1963	10.0% (6.5-14.1)	959.17	< 0.01	95.6%	0.001	0.119 (0.049 to 0.189)
	30-60°W	5	2755	134	8.4% (2.1-18.2)	95.14	< 0.01	95.8%		

	60-90°E	17	7524	185	3.5% (2.0-5.3)	135.02	< 0.01	88.1%		
	90-120°E	20	32790	241	4.8% (1.6-9.2)	879.54	< 0.01	97.8%		
Latitude										
	0-20°S	21	2944	102	3.0% (0.0-8.8)	88.51	< 0.01	77.4%		
	0-30°N	21	33436	263	4.2% (1.5-8.0)	951.20	< 0.01	97.9%		
	20-30°S	27	12426	2040	16.0% (12.4-20.0)	736.74	< 0.01	96.5%	<.0001	0.186 (0.118 to 0.253)
	30-60°N	25	225552	5309	4.3% (2.5-6.5)	15285.51	0	99.8%		
Climate										
	Mediterranean climate	10	220592	5162	3.2% (0.9-6.5)	15056.79	0	99.9%		
	Tropical savanna climate	54	59318	5045	10.1% (7.2-13.6)	3012.32	0	98.2%	<.0001	0.119 (0.063 to 0.175)
	Tropical monsoon climate	12	5932	551	7.5% (3.8-12.3)	240.31	< 0.01	95.4%		
	Tropical desert climate	15	6611	167	3.8% (2.3-5.7)	108.70	< 0.01	87.1%		
	Tropical rainforest climate	12	9268	437	6.8% (3.1-11.6)	292.36	< 0.01	96.2%		
	Temperate continental climate	1	188	15	8.0% (4.5-12.3)	0.00	- -	- -		
	Subtropical monsoon climate	19	32701	224	4.3% (1.3-8.6)	816.43	< 0.01	97.8%		
Region										
	Africa	39	19635	2290	10.1% (6.9-13.7)	1725.02	0	97.8%	0.022	0.077 (0.011 to 0.143)
	Asia	47	93436	1174	6.0% (3.9-8.6)	3743.94	0	98.8%		
	Europe	3	217689	5081	2.6% (0.0-15.3)	14952.94	0	100.0%		
	Oceania	3	45567	2932	4.6% (1.5-9.1)	830.45	< 0.01	99.8%		
	South America	12	9289	440	7.1% (3.2-12.4)	275.28	< 0.01	96.0%		
Subregion										
	East Asia	20	79873	409	6.4% (2.2-12.4)	1473.46	< 0.01	98.7%		
	Eastern Africa	8	501	62	13.5% (3.9-27.1)	78.68	< 0.01	91.1%		
	North Africa	9	5565	114	2.7% (1.2-4.6)	65.23	< 0.01	87.7%		
	Oceania	3	45567	2932	4.6% (1.5-9.1)	830.45	< 0.01	99.8%		
	South America	12	9289	440	7.1% (3.2-12.4)	275.28	< 0.01	96.0%		

	South Asia	25	12870	741	5.7% (3.9-7.9)	608.74	< 0.01	96.1%		
	Southeast Asia	2	693	24	7.4% (0.0-33.5)	37.14	< 0.01	97.3%		
	Southern Africa	22	13569	2114	13.0% (8.8-17.8)	508.02	< 0.01	95.9%	0.003	0.116 (0.040 to 0.192)
	Southern Europe	3	217689	5081	2.6% (0.0-15.3)	14952.94	0	100.0%		
Development status										
	Developed country	6	263256	8013	3.6% (0.5-9.1)	19733.06	0	100.0%	0.157	-0.097 (-0.232 to 0.037)
	Developing country	89	120671	3810	7.6% (5.8-9.6)	10405.18	0	99.2%		
	Landlocked developing country	9	1689	94	9.7% (1.8-21.8)	123.69	< 0.01	93.5%		
Income level										
	High-income economy	6	263256	8013	3.6% (0.5-9.1)	19733.06	0	100.0%		
	Upper-middle-income economy	51	102624	2975	9.7% (6.8-13.1)	9001.75	0	99.4%		
	Lower-middle-income economy	42	19421	884	4.8% (3.5-6.4)	827.40	< 0.01	95.0%	0.032	-0.072 (-0.137 to -0.006)
	Low-income economy	5	315	45	13.7% (0.0-40.6)	82.79	< 0.01	95.2%		
BCS										
	Good	7	1143	37	4.5% (0.4-11.3)	30.15	< 0.01	80.1%		
	Poor	2	227	18	33.3% (0.1-99.8)	24.37	< 0.01	95.9%	0.166	0.284 (-0.118 to 0.685)
Gender										
	Female	22	16501	671	5.7% (3.3-8.5)	383.01	< 0.01	94.5%	0.415	0.046 (-0.065 to 0.157)
	Male	11	6621	137	3.5% (0.0-12.6)	52.91	< 0.01	81.1%		
Age										
	< 1	5	51	0	0.00% (0.0-0.4)	0.63	0.96	0.0%		
	1-5	14	1011	32	0.5% (0.0-3.7)	77.48	< 0.01	83.2%		
	5-15	14	2622	265	11.7% (4.7-21.0)	245.74	< 0.01	94.7%		
	15-20	3	30	9	34.2% (2.6-75.4)	9.76	< 0.01	79.5%	0.018	0.359 (0.061 to 0.656)
Weight (kg)										
	≤ 500	6	1694	66	3.4% (1.2-6.6)	28.41	< 0.01	82.4%		
	> 500	5	988	98	9.9% (4.0-17.8)	39.50	< 0.01	89.9%	0.047	0.132 (0.002 to 0.262)

Milk Yield (Liters)										
	< 5	5	648	14	1.5% (0.5-2.8)	5.19	0.27	23.0%		
	5-10	5	1489	76	4.1% (2.2-6.6)	16.01	< 0.01	75.0%		
	10-15	3	690	69	7.3% (0.0-30.2)	91.14	< 0.01	97.8%	0.450	0.070 (-0.111 to 0.250)
	> 15	2	50	4	9.3% (0.0-59.8)	11.34	< 0.01	91.2%		
Parity										
	< 5	5	1740	81	4.6% (2.4-7.5)	24.08	< 0.01	83.4%	0.152	-0.101 (-0.240 to 0.037)
	5-10	2	348	34	11.0% (0.5-31.3)	20.88	< 0.01	95.2%		
	> 10	1	44	3	6.8% (0.9-16.6)	0.00	- -	- -		
Herd size										
	< 100	6	1087	32	0.0% (0.0-0.0)	8.91	0.11	43.9%	<.0001	-0.160 (-0.216 to -0.105)
	100-1000	6	678	81	10.3% (4.8-17.1)	12.17	0.03	58.9%		
	> 1000	1	406	40	9.9% (7.1-13.0)	0.00	- -	- -		
Purpose										
	Dairy buffalo	32	88432	946	4.1% (2.6-5.9)	3022.88	0	99.0%	<.0001	-0.177 (-0.249 to -0.105)
	Draught buffalo	4	219	78	28.0% (15.3-42.6)	8.19	0.04	63.4%		
	Wild buffalo	29	13795	2121	12.7% (8.6-17.4)	591.69	< 0.01	95.3%		
Source										
	Abattoir	15	238467	3315	3.5% (1.3-6.7)	8054.09	0	99.8%		
	Farm	31	63044	1100	5.9% (4.0-8.1)	3043.38	0	99.0%		
	Free-ranging	30	13875	2121	12.0% (8.0-16.7)	628.05	< 0.01	95.4%	0.001	0.117 (0.051 to 0.182)
	Rural/Village	7	2394	103	6.3% (2.3-11.8)	39.04	< 0.01	84.6%		
Detection method										
	Culture	8	4443	105	5.7% (1.5-12.1)	202.01	< 0.01	96.5%	0.577	-0.036 (-0.162 to 0.090)
	IFN-γ-ELISA	23	37501	5248	9.1% (5.0-14.2)	1128.07	< 0.01	98.0%		
	PCR	25	11979	249	6.8% (3.2-11.5)	743.70	< 0.01	96.8%		
	PM	18	242011	3960	8.3% (3.8-14.2)	9924.25	0	99.8%		

	Radiodiagnosis	1	217	24	11.1% (7.2-15.6)	0.00	- -	- -		
	SMS	1	159	6	3.8% (1.3-7.4)	0.00	- -	- -		
	TB ELISA	9	2131	193	6.2% (2.3-11.5)	152.73	< 0.01	94.8%		
	TST	62	107791	2811	7.8% (5.6-10.3)	8170.83	0	99.3%		
	ZN	4	1430	200	10.1% (5.5-15.7)	26.56	< 0.01	88.7%		
Tuberculin test										
	SITT	30	84968	596	6.1% (3.1-10.0)	2029.91	0	98.6%	0.133	-0.067 (-0.155 to 0.021)
	CITT	30	21737	2160	9.9% (6.8-13.5)	1375.43	< 0.01	97.9%		
Sample										
	Bacterial colonies	4	514	48	9.1% (6.7-11.9)	1.46	0.69	0.0%		
	Blood	25	37761	5302	9.8% (5.1-15.6)	1281.75	< 0.01	98.1%		
	Fecal	1	26	2	7.7% (0.2-21.8)	0.00	- -	- -		
	Milk	8	669	48	8.6% (2.8-16.6)	40.30	< 0.01	82.6%		
	Serum	9	2131	193	6.2% (2.3-11.5)	152.73	< 0.01	94.8%		
	TB-like lesions	6	12881	58	1.3% (0.0-5.0)	113.28	< 0.01	95.6%	0.011	-0.175 (-0.311 to -0.040)
	Tissue (Lymph nodes)	17	3661	350	7.1% (3.9-11.0)	212.32	< 0.01	92.5%		
Species										
	M.bovis	17	5366	193	7.4% (3.0-13.3)	582.43	< 0.01	97.3%		
	M.orygis	2	3205	16	3.3% (0.0-20.7)	21.38	< 0.01	95.3%	0.545	-0.089 (-0.379 to 0.200)
	M.tuberculosis	5	3377	29	3.5% (0.0-12.0)	67.86	< 0.01	94.1%		
	MTBC	1	31	11	35.5% (19.4-53.3)	0.00	- -	- -		
Quality level										
	2-3	44	307394	4095	7.0% (4.5-10.0)	11555.66	0	99.6%	0.719	-0.012 (-0.078 to 0.054)
	4-5	60	78222	7822	7.6% (5.5-10.0)	5599.79	0	98.9%		
Total		104	385616	11917	7.4% (5.7-9.2)	30329.77	0	99.7%		

CI: Confidence interval. BCS: Body condition score. Region: Oceania: Australia; Africa: Botswana, Egypt, Mozambique, South Africa, Tanzania, Uganda, Zambia, Zimbabwe; South America: Brazil; Asia: China ,India, Lao PDR, Nepal, Pakistan, Thailand; Europe: Italy. Subregion: East Asia: China; South Asia: Pakistan, India, Nepal; Southeast Asia: Lao PDR, Thailand; Eastern Africa:

Tanzania, Uganda; North Africa: Egypt; Southern Africa: South Africa, Mozambique, Zambia, Botswana, Zimbabwe; Oceania: Australia; South America: Brazil; Southern Europe: Italy. Detection method: IFN-γ-ELISA: Interferon-γ-enzyme linked immunosorbent assay; PCR: Polymerase chain reaction; PM: Post mortem; SMS: Shotgun metagenomic sequencing; TB ELISA: Bovine tuberculosis enzyme linked immunosorbent assay; TST: Tuberculin skin test; ZN: Ziehl-Neelsen.Tuberculin test: SITT: Single intradermal tuberculin test; CITT: Comparative intradermal tuberculin test. Species: *M.bovis*: *Mycobacterium bovis*; *M.orygis*: *Mycobacterium orygis*; *M.tuberculosis*: *Mycobacterium tuberculosis*; MTBC: *Mycobacterium tuberculosis* complex.

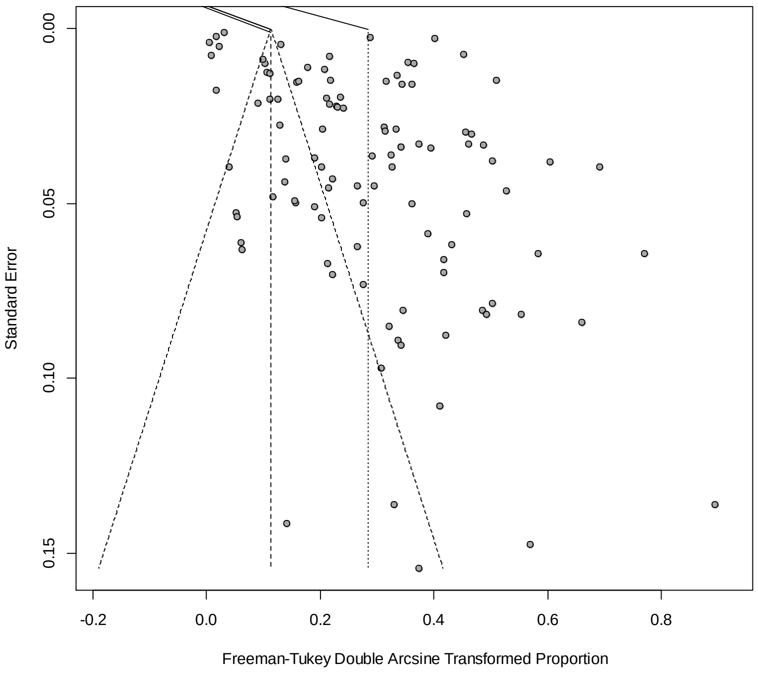

Figure S1. Funnel plot with pseudo 95% confidence intervals for examining publication bias in the publication years subgroup of global buffalo tuberculosis.

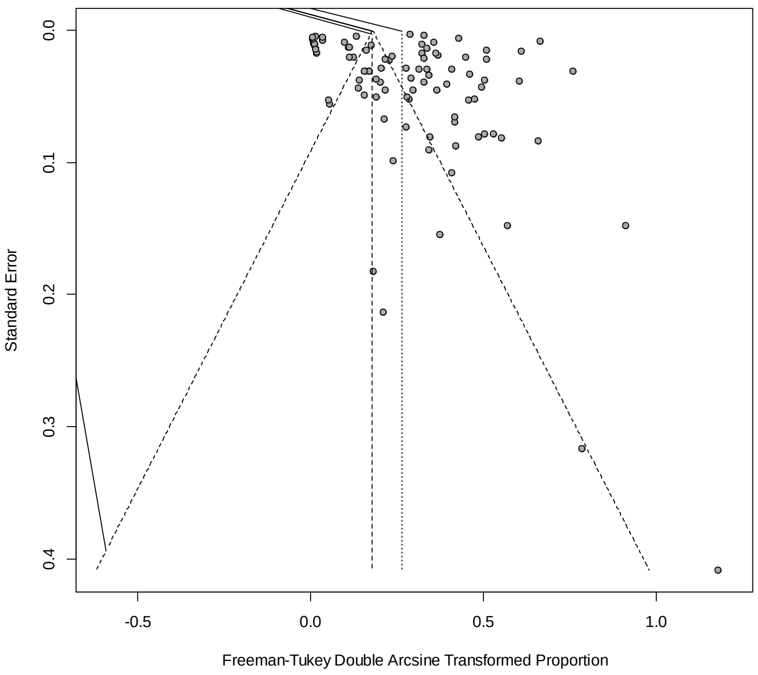

Figure S2. Funnel plot with pseudo 95% confidence intervals for examining publication bias in the sampling years subgroup of global buffalo tuberculosis.

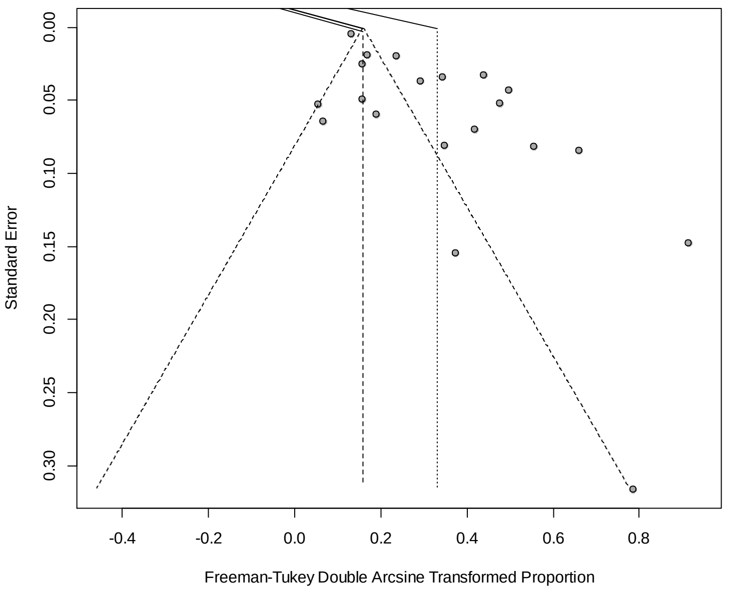

Figure S3. Funnel plot with pseudo 95% confidence intervals for examining publication bias in the season subgroup of global buffalo tuberculosis.

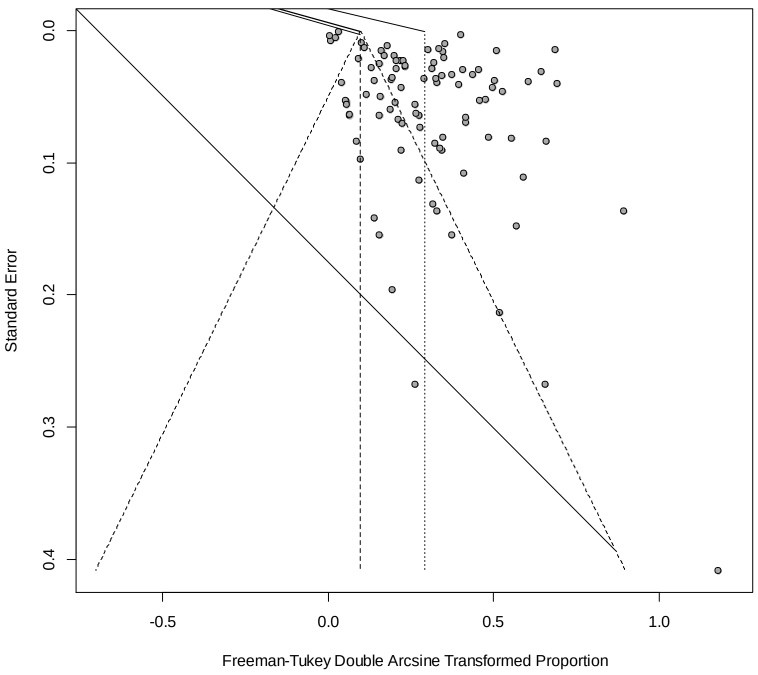

Figure S4. Funnel plot with pseudo 95% confidence intervals for examining publication bias in the longitude subgroup of global buffalo tuberculosis.

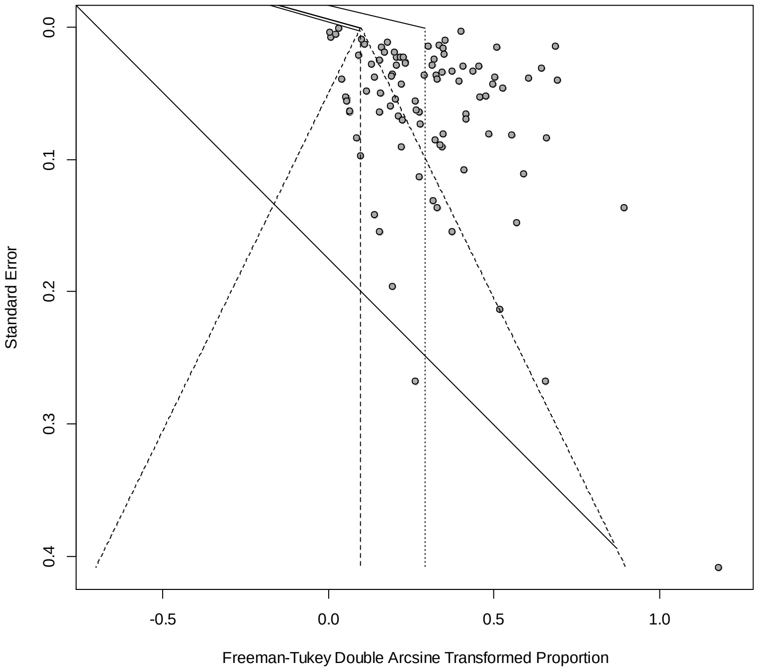

Figure S5. Funnel plot with pseudo 95% confidence intervals for examining publication bias in the latitude subgroup of global buffalo tuberculosis.

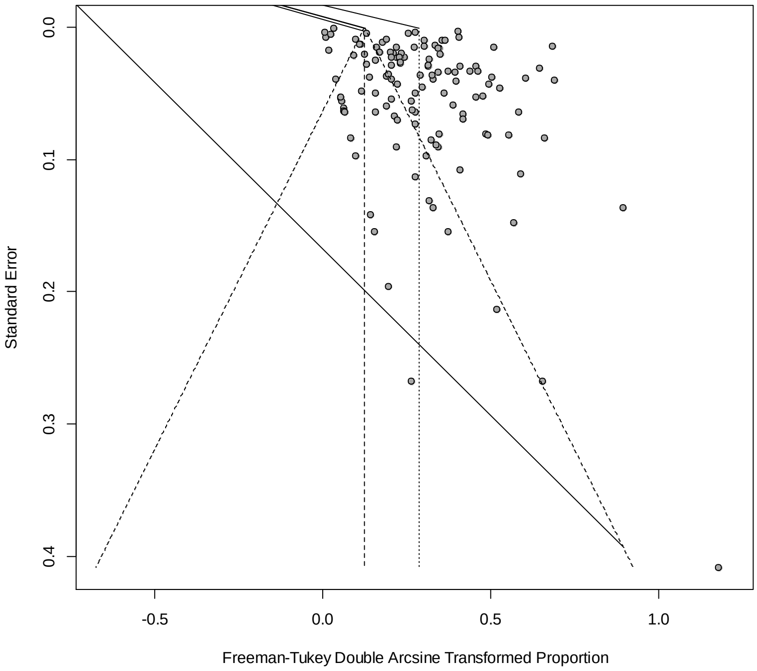

Figure S6. Funnel plot with pseudo 95% confidence intervals for examining publication bias in the climate subgroup of global buffalo tuberculosis.

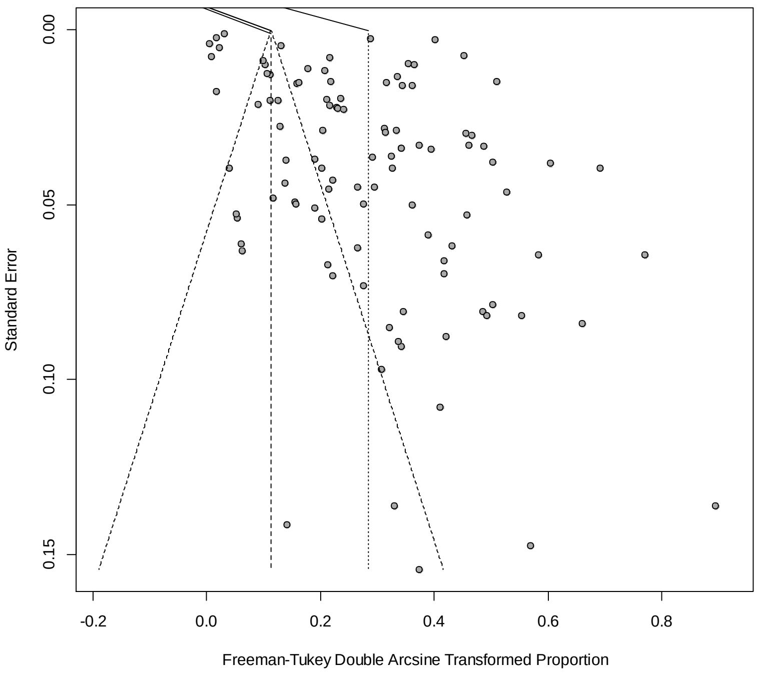

Figure S7. Funnel plot with pseudo 95% confidence intervals for examining publication bias in the region subgroup of global buffalo tuberculosis.

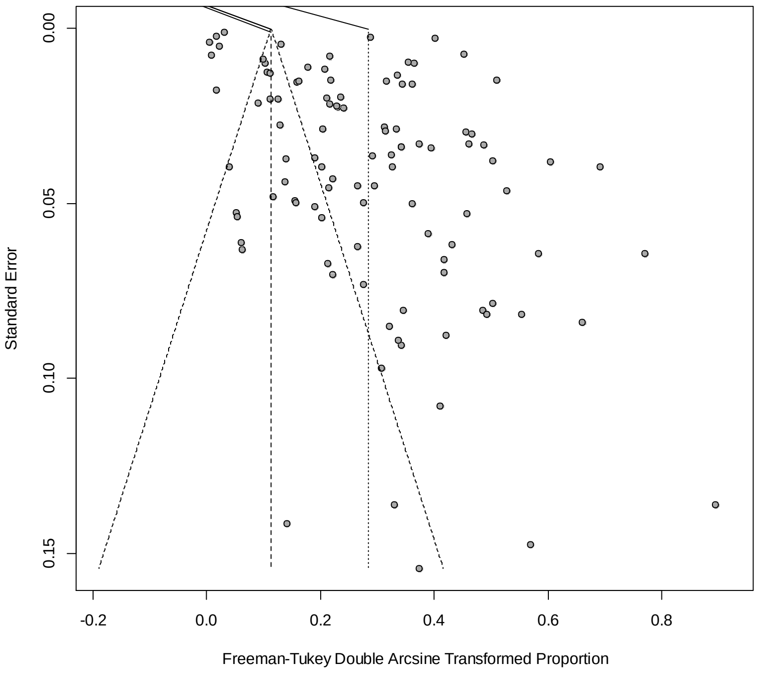

Figure S8. Funnel plot with pseudo 95% confidence intervals for examining publication bias in the subregion subgroup of global buffalo tuberculosis.

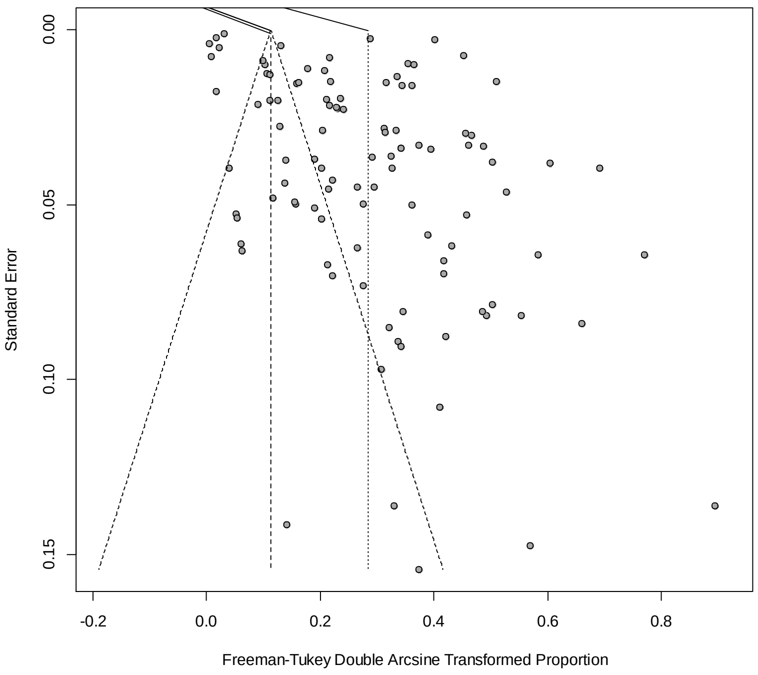

Figure S9. Funnel plot with pseudo 95% confidence intervals for examining publication bias in the development status subgroup of global buffalo tuberculosis.

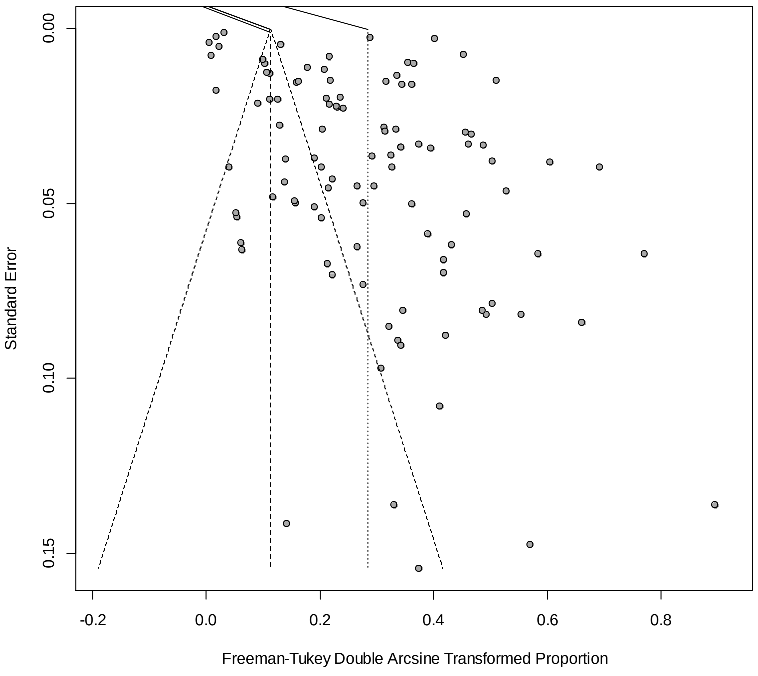

Figure S10. Funnel plot with pseudo 95% confidence intervals for examining publication bias in the income level subgroup of global buffalo tuberculosis.

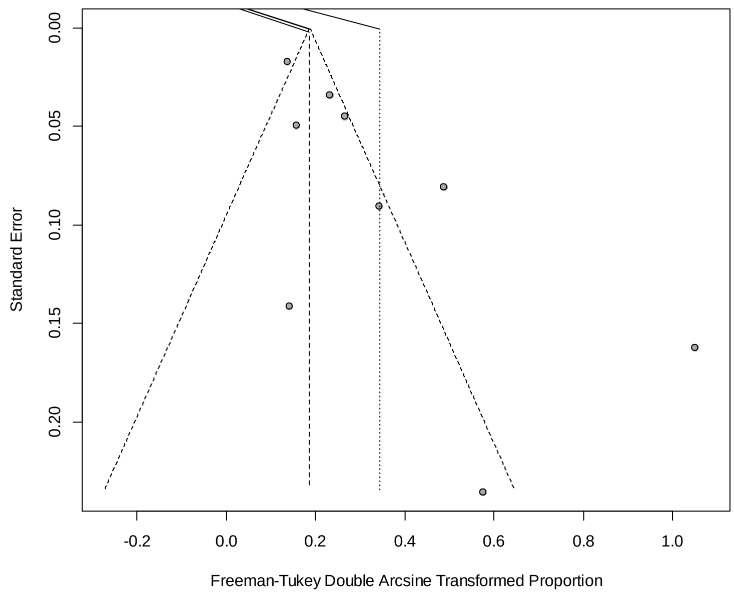

Figure S11. Funnel plot with pseudo 95% confidence intervals for examining publication bias in the BCS subgroup of global buffalo tuberculosis.

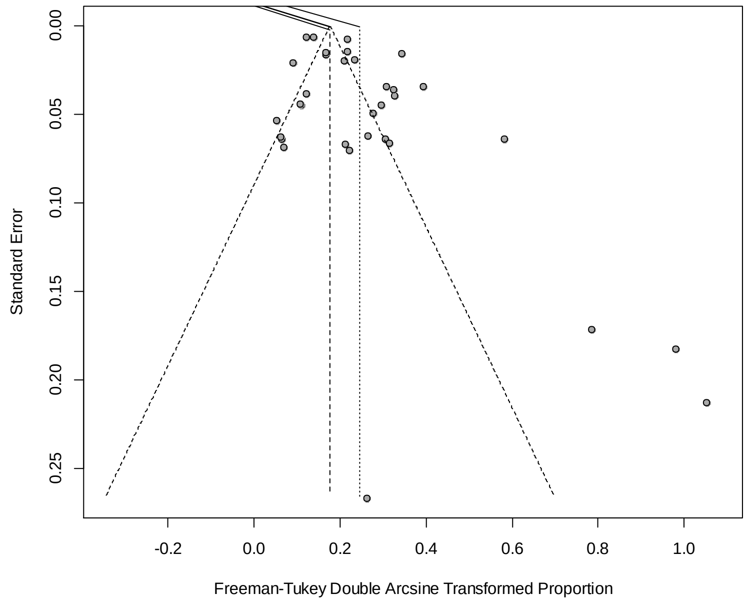

Figure S12. Funnel plot with pseudo 95% confidence intervals for examining publication bias in the gender subgroup of global buffalo tuberculosis.

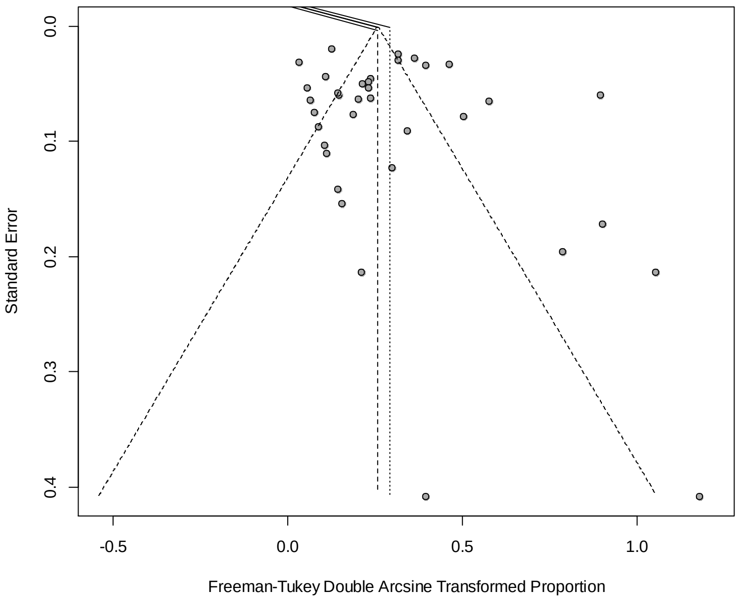

Figure S13. Funnel plot with pseudo 95% confidence intervals for examining publication bias in the age subgroup of global buffalo tuberculosis.

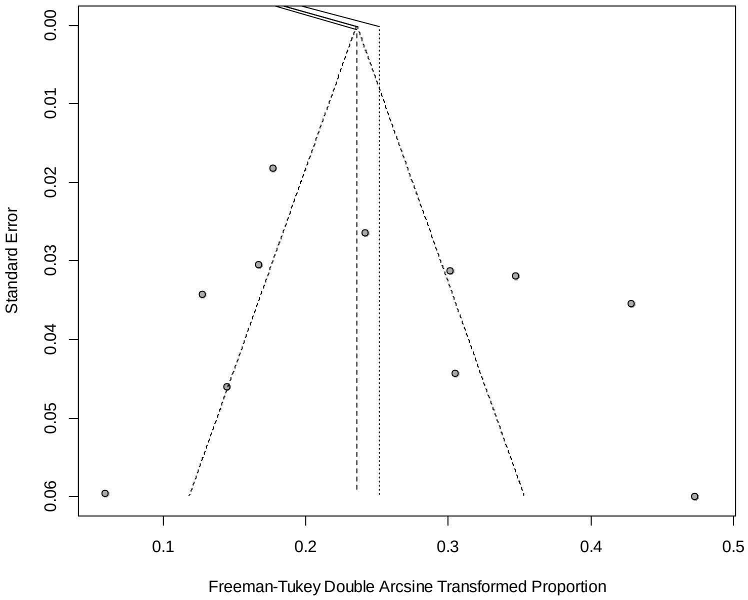

Figure S14. Funnel plot with pseudo 95% confidence intervals for examining publication bias in the weight (kg) subgroup of global buffalo tuberculosis.

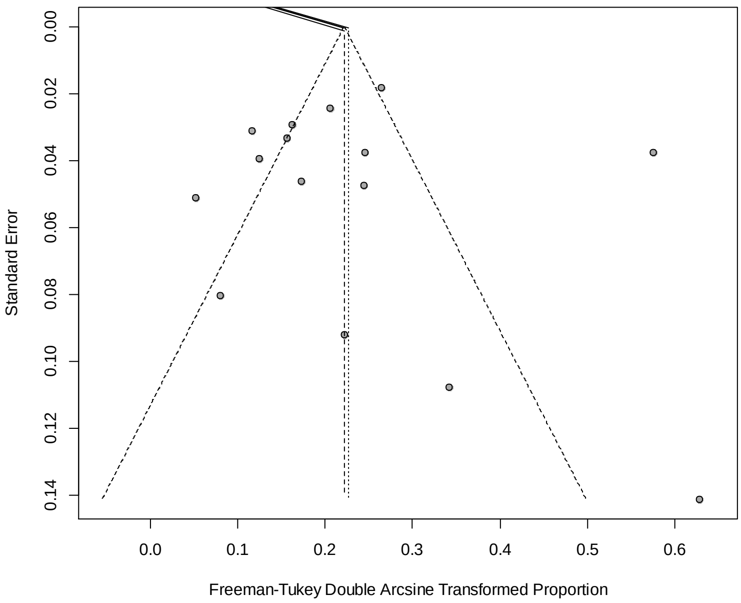

Figure S15. Funnel plot with pseudo 95% confidence intervals for examining publication bias in the milk yield (Liters) subgroup of global buffalo tuberculosis.

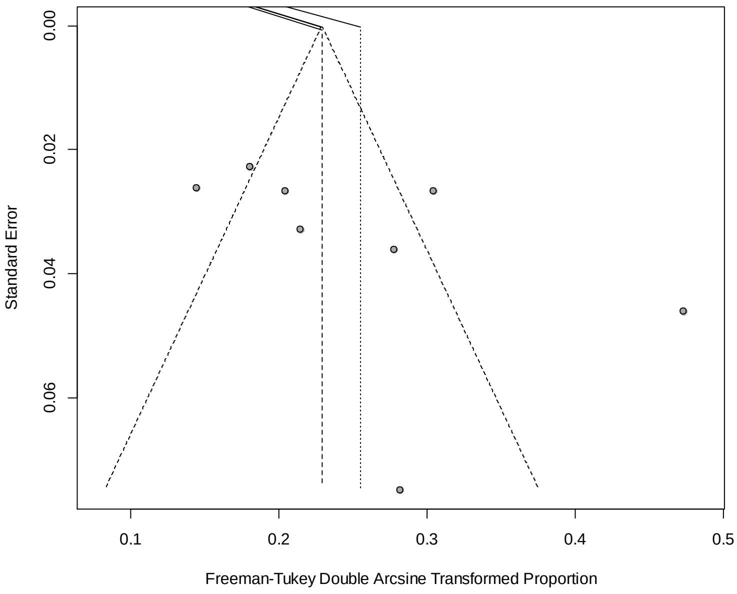

Figure S16. Funnel plot with pseudo 95% confidence intervals for examining publication bias in the parity subgroup of global buffalo tuberculosis.

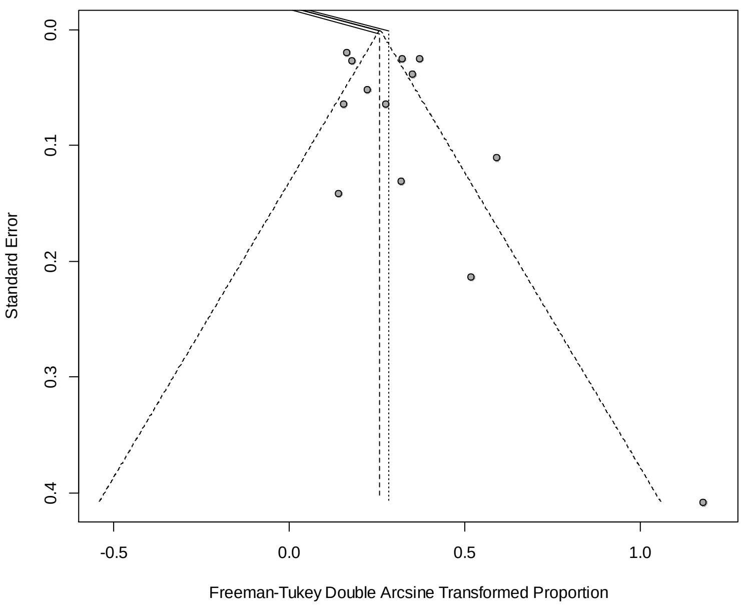

Figure S17. Funnel plot with pseudo 95% confidence intervals for examining publication bias in the herd size subgroup of global buffalo tuberculosis.

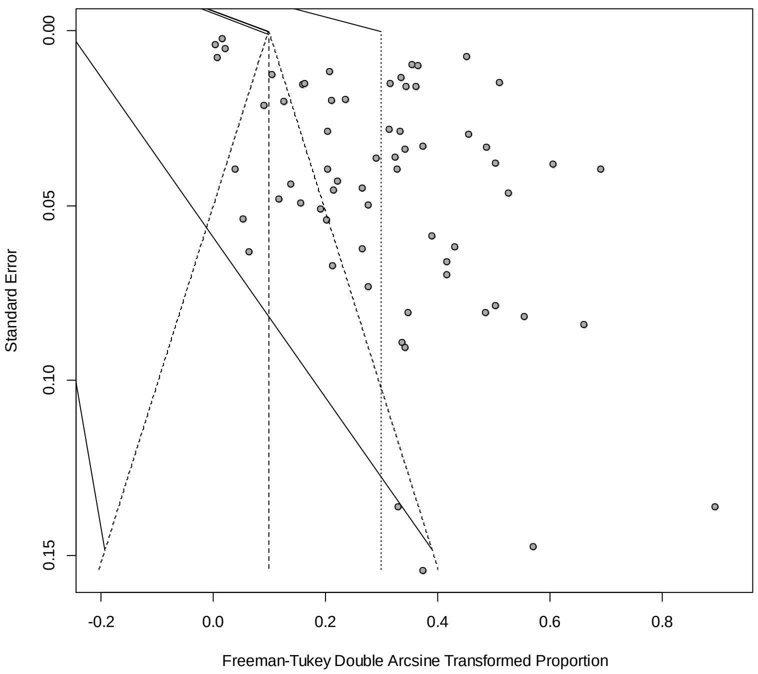

Figure S18. Funnel plot with pseudo 95% confidence intervals for examining publication bias in the purpose subgroup of global buffalo tuberculosis.

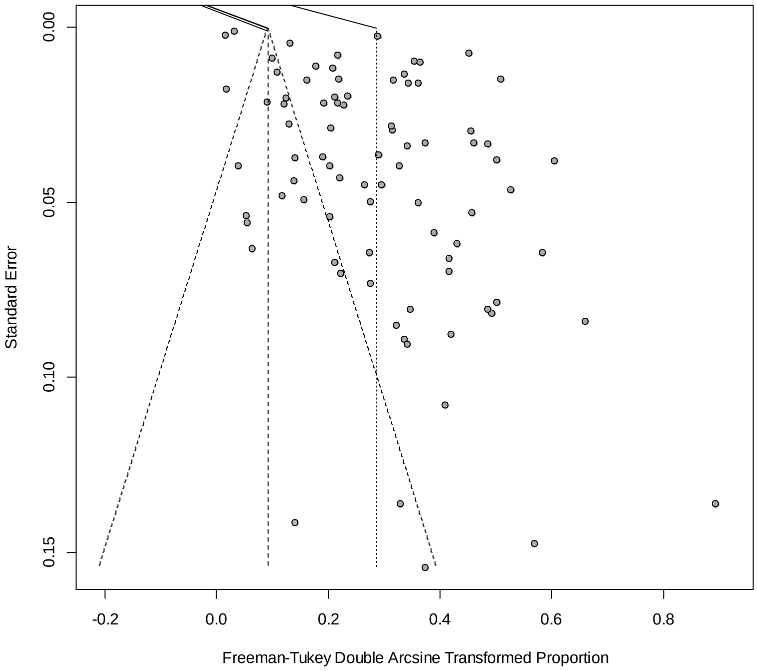

Figure S19. Funnel plot with pseudo 95% confidence intervals for examining publication bias in the source subgroup of global buffalo tuberculosis.

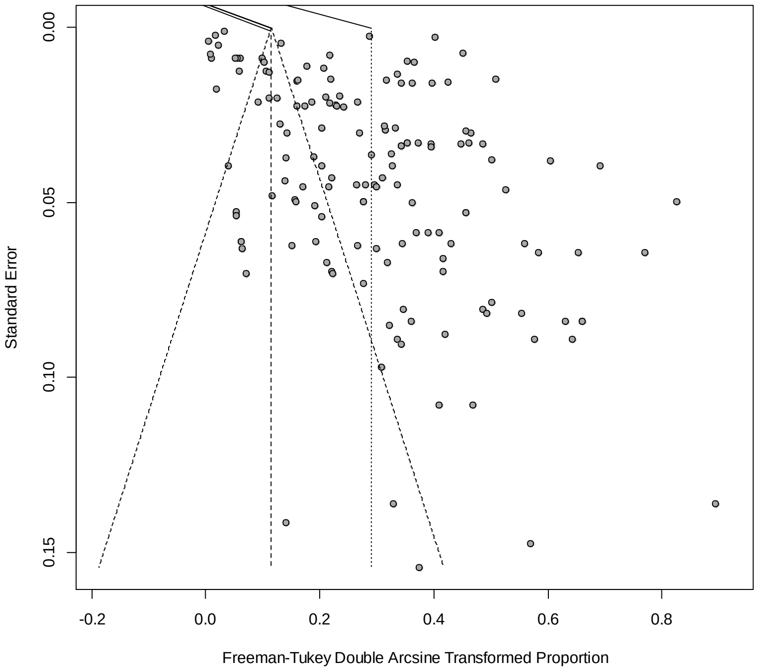

Figure S20. Funnel plot with pseudo 95% confidence intervals for examining publication bias in the detection method subgroup of global buffalo tuberculosis.

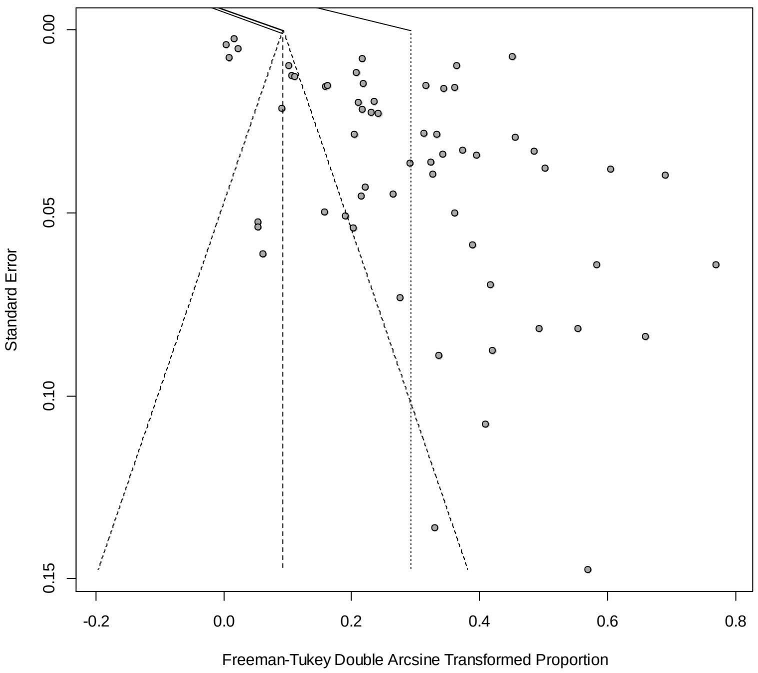

Figure S21. Funnel plot with pseudo 95% confidence intervals for examining publication bias in the tuberculin test subgroup of global buffalo tuberculosis.

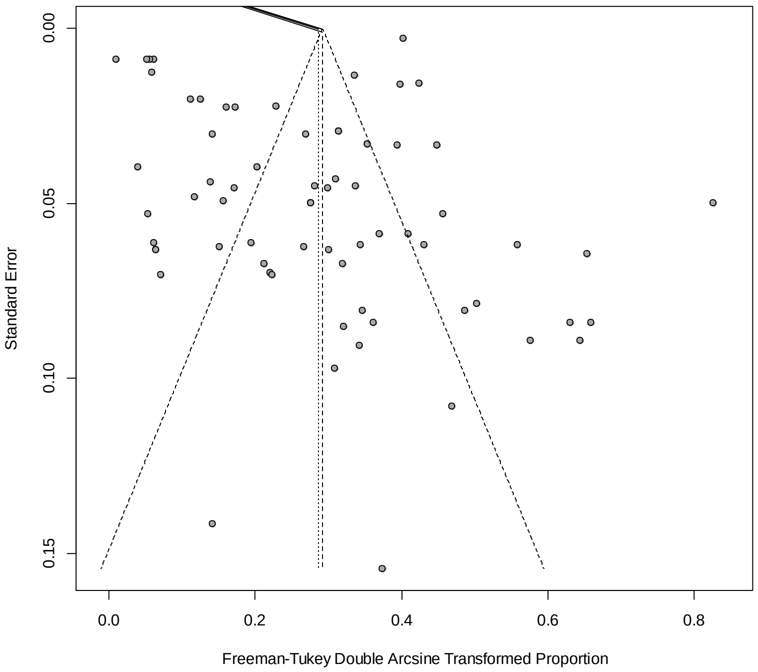

Figure S22. Funnel plot with pseudo 95% confidence intervals for examining publication bias in the sample subgroup of global buffalo tuberculosis.

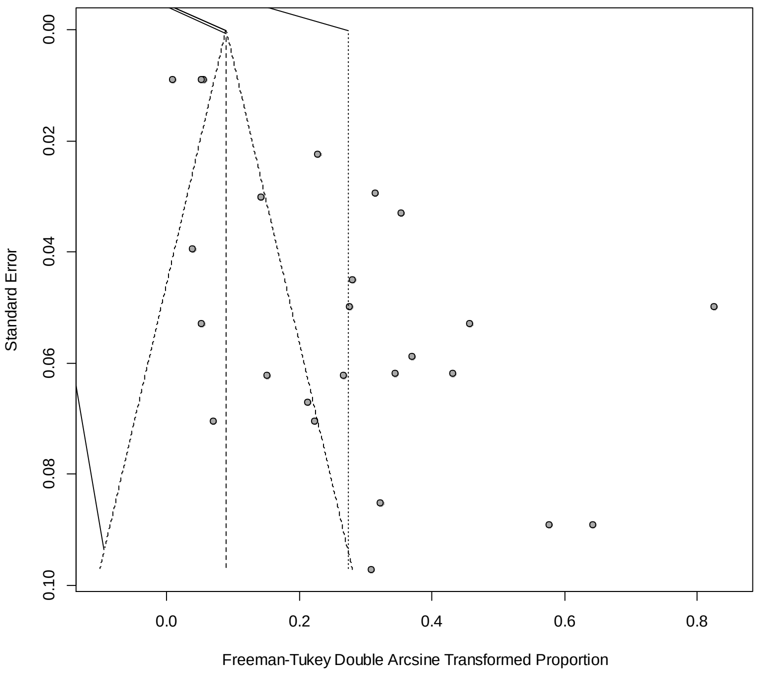

Figure S23. Funnel plot with pseudo 95% confidence intervals for examining publication bias in the species subgroup of global buffalo tuberculosis.

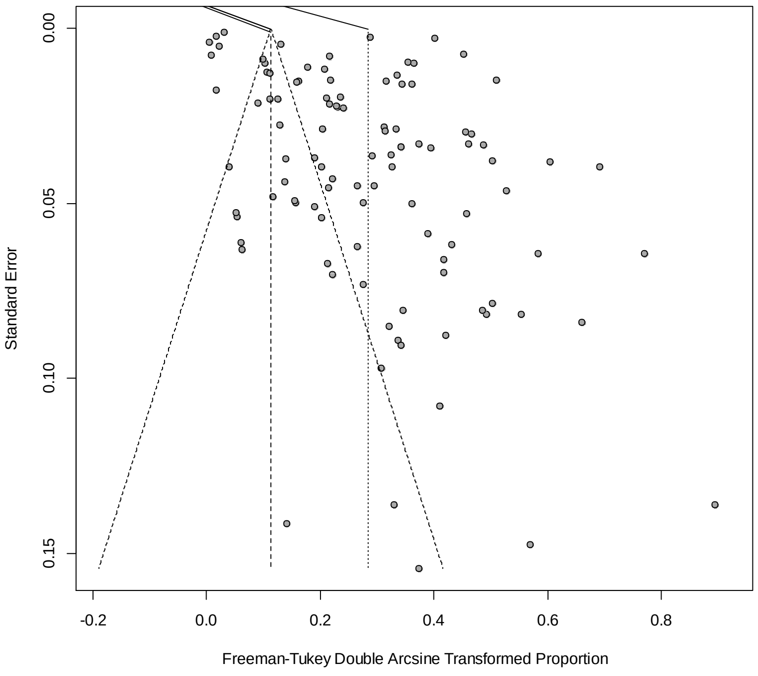

Figure S24. Funnel plot with pseudo 95% confidence intervals for examining publication bias in the quality level subgroup of global buffalo tuberculosis.
